# Progress on hydrogel delivery systems targeting metabolism disorders in the treatment of diabetic foot ulcers

**DOI:** 10.3389/fcell.2026.1860656

**Published:** 2026-09-04

**Authors:** Shihua Liu, Lixian Huang, Hong Dong

**Affiliations:** Department of Orthopedic Surgery, West China Hospital, West China School of Nursing, Sichuan University, Chengdu, Sichuan, China

**Keywords:** diabetic foot ulcer, endothelial cells, fibroblasts, macrophages, metabolism

## Abstract

Diabetic foot ulcer (DFU) is a severe and difficult-to-heal complication of diabetes in which systemic metabolic dysregulation is translated into local neurovascular injury and dysfunction of wound-healing cells. This narrative review examines the relationships among glucose, lipid, and amino acid/protein metabolism disorders, the dysfunction of macrophages, fibroblasts, and endothelial cells, and hydrogel-based therapeutic strategies. Persistent hyperglycemia disrupts the tricarboxylic acid cycle, activates the polyol and advanced glycation pathways, and induces pseudohypoxia, oxidative stress, and inflammatory metabolic reprogramming. Dyslipidemia promotes lipid peroxidation, mitochondrial injury, impaired fatty acid oxidation, and ferroptosis, whereas amino acid and protein metabolic abnormalities disturb insulin signaling, arginine metabolism, collagen-precursor availability, and extracellular matrix turnover. These metabolic alterations sustain pro-inflammatory macrophage programs, impair fibroblast migration and matrix production, and compromise endothelial angiogenesis. We further critically compare hydrogel systems that provide glucose-responsive insulin release, catalytic glucose consumption and oxygen generation, lipid-peroxide scavenging, immunometabolic regulation, and support for extracellular matrix remodeling and vascular regeneration. Metabolism-targeted hydrogels provide a promising bridge between mechanistic metabolic intervention and local wound management; however, most available evidence remains preclinical. Future translation requires direct validation of metabolic regulation, standardized manufacturing, long-term biosafety assessment, and evaluation in clinically relevant DFU models.

## 1 Introduction

Diabetic foot ulcer (DFU) is one of the serious complications in diabetic patients, characterized by deep tissue damage caused by foot infections, vascular abnormalities, and neurological disorders (Armstrong et al., 2023). According to statistics, the global prevalence of diabetes was estimated at 537 million people in 2021 and is projected to rise to 783.2 million by 2045 (Sun et al., 2022). The incidence of DFU ranges from 19% to 34% (Armstrong et al., 2017; Wang et al., 2024a), and the rate of non-traumatic lower extremity amputations reaches 20% (Zhang et al., 2020).

DFU refers to infections, ulcers, and other injuries occurring in tissues distal to the ankle in diabetic patients, which are associated with lower extremity neuropathy and peripheral arterial disease (Armstrong et al., 2023). It is a common and severe complication in diabetic patients, resulting from a combination of neuropathic, vascular, and biomechanical factors. This complication increases the risk of soft tissue infections, peripheral arterial disease, and mortality (Armstrong et al., 2023). In addition to risk factors such as loss of protective sensation, foot deformities, secondary ulcers, infections, vascular lesions, and polyneuropathy, the occurrence of DFU is closely related to energy metabolism disorders. Energy metabolism disorder refers to disruptions in key energy-producing processes in the body and cells, including the uptake, transport, and oxidative utilization of energy substrates such as glucose, as well as mitochondrial oxidative phosphorylation. It involves interactions among various biomolecules and signaling pathways (Wu et al., 2025; Pan et al., 2025).

Traditional methods for treating DFU, such as debridement, offloading, and the use of conventional dressings (e.g., gauze), although essential components of basic care, often struggle to effectively address the complex pathological challenges mentioned above (Huang et al., 2023; Feng et al., 2023; Liu et al., 2024). An ideal wound dressing should provide a moist healing environment, effectively control infection, absorb exudate, and actively promote tissue regeneration. However, conventional dressings offer relatively limited functionality. Therefore, it is crucial to develop novel therapeutic strategies and advanced biomaterials that can target the pathological microenvironment of DFU.

Hydrogels, which are three-dimensional network-structured polymer materials, exhibit excellent biocompatibility and drug delivery capabilities, and can adsorb and retain large amounts of exudate (Mallanagoudra et al., 2025; Du et al., 2026). By incorporating or loading different substances, hydrogels can acquire new chemical properties, showing promising application prospects in the treatment of DFU (Jin et al., 2025).

This review summarizes the relationship between energy substrate metabolism disorders and DFU progression, the effects of metabolic abnormalities on key wound-healing cells, and recent advances in hydrogel delivery systems targeting metabolic dysfunction for DFU treatment.

## 2 Methodology

This narrative review was prepared using a structured literature-search strategy to improve the transparency and reproducibility of study selection. Electronic searches were performed in PubMed/MEDLINE, Web of Science, Scopus, and Embase from January 2010 to April 2026. Additional relevant articles were identified by manually screening the reference lists of key reviews and original research articles.

The search strategy combined terms related to diabetic foot ulcers, hydrogel-based delivery systems, metabolic disorders, and wound-cell dysfunction. Representative search terms included: “diabetic foot ulcer”, “diabetic wound”, “chronic diabetic wound”, “hydrogel”, “injectable hydrogel”, “smart hydrogel”, “responsive hydrogel”, “wound dressing”, “drug delivery”, “metabolic disorder”, “metabolic reprogramming”, “glucose metabolism”, “glycolysis”, “tricarboxylic acid cycle”, “polyol pathway”, “lipid metabolism”, “fatty acid oxidation”, “amino acid metabolism”, “protein metabolism”, “mitochondrial dysfunction”, “oxidative stress”, “AGE-RAGE”, “macrophage polarization”, “fibroblast dysfunction”, and “endothelial cell dysfunction”. These terms were combined using Boolean operators. A representative search string was: (“diabetic foot ulcer” OR “diabetic wound” OR “chronic diabetic wound”) AND (“hydrogel” OR “injectable hydrogel” OR “responsive hydrogel” OR “wound dressing” OR “drug delivery”) AND (“metabolism” OR “metabolic reprogramming” OR “glucose metabolism” OR “lipid metabolism” OR “amino acid metabolism” OR “mitochondrial dysfunction” OR “oxidative stress” OR “macrophage” OR “fibroblast” OR “endothelial cell”).

Because this article is a narrative review rather than a systematic review or meta-analysis, representative studies were selected based on their mechanistic relevance, novelty of hydrogel design, quality of experimental evidence, and translational significance. Particular attention was given to hydrogel systems that directly modulate metabolic abnormalities, including glucose-responsive delivery, glucose-consuming catalytic systems, lipid peroxide clearance, fatty acid oxidation regulation, amino acid/protein metabolic intervention, macrophage metabolic reprogramming, fibroblast functional restoration, and endothelial metabolic protection. The final selection was organized according to two complementary dimensions: major metabolic substrate disorders, including glucose, lipid, and protein metabolism, and metabolism-related dysfunction of key wound-healing cells, including macrophages, fibroblasts, and endothelial cells.

For the representative hydrogel systems discussed in this review, key information on hydrogel composition, delivered cargo, stimulus-responsive behavior, characterization evidence, experimental model, and therapeutic outcome was extracted from the original studies and summarized where relevant. Detailed preparation parameters, analytical protocols, sample sizes, and statistical methods should be referred to in the corresponding original research articles.

## 3 Molecular mechanisms underlying the association between energy metabolism disorders and DFU

Energy metabolism refers to all reactions involved in the generation of ATP from nutrients, including aerobic oxidation of carbohydrates, anaerobic respiration, and the metabolism of fatty acids and amino acids. As a common complication of diabetes, the occurrence and progression of DFU are closely associated with diabetic lower extremity neuropathy and peripheral vascular disease (PVD). According to statistics, over 60% of DFU cases result from underlying neuropathy, and PVD plays a significant role in the progression of the condition in up to 50% of DFU patients (Sattaputh et al., 2012). Autonomic neuropathy can lead to impaired vasomotor regulation in the feet, as well as reduced function of sweat and sebaceous glands (Sattaputh et al., 2012); motor neuropathy can cause atrophy of foot muscles, foot deformities, and increased plantar skin pressure (Pop-Busui et al., 2022); sensory neuropathy results in a lack of protective sensation in the feet, preventing timely perception of injurious stimuli (Bandyk, 2018); PVD can directly reduce blood flow to the lower extremities and cause local tissue ischemia, thereby triggering ulcers and impairing wound healing (Singh et al., 2005). Energy metabolism disorders in diabetic patients involve multiple levels, including abnormalities in glucose, lipid, and protein metabolism, all of which are closely linked to diabetic neuropathy and PVD.

### 3.1 Disorders of glucose metabolism

Persistent hyperglycemia contributes to the initiation and progression of DFU through two closely interconnected metabolic routes: dysfunction of central glucose oxidation and excessive activation of the polyol pathway. These pathways jointly impair mitochondrial energy production, disrupt cellular redox homeostasis, promote inflammatory metabolic reprogramming, and damage peripheral nerves and vascular endothelial cells. Consequently, glucose metabolism disorder not only increases the susceptibility of diabetic skin to ulceration but also creates a local microenvironment that prevents normal wound repair.

Under physiological conditions, glucose is metabolized through glycolysis, the tricarboxylic acid (TCA) cycle, and mitochondrial oxidative phosphorylation to generate ATP. The TCA cycle also connects glucose metabolism with lipid and amino acid metabolism and therefore functions as a central metabolic hub (Arnold and Finley, 2023). In diabetic wounds, metabolomic studies have identified abnormalities in TCA-cycle and pyruvate-related metabolites, including altered levels of citrate, cis-aconitate, malate, lactate, and succinate (Jiang et al., 2023; Wang and Hu, 2025; Li et al., 2023). At the cellular level, metabolic breakpoints at isocitrate dehydrogenase and succinate dehydrogenase impair continuous TCA-cycle flux and promote the accumulation of inflammatory metabolites such as citrate and succinate (O'Neill and Artyomov, 2019). Reduced mitochondrial oxidative metabolism forces cells to rely more strongly on glycolysis for ATP generation. In macrophages, this glycolysis-dominant state, together with succinate accumulation and excessive mitochondrial ROS production, reinforces pro-inflammatory signaling and prevents the timely acquisition of repair-associated functions. Persistent macrophage activation subsequently prolongs inflammation, increases tissue damage, and delays the transition from the inflammatory phase to the proliferative phase of wound healing.

In parallel, chronic hyperglycemia increases the entry of glucose into the polyol pathway. Aldose reductase converts glucose into sorbitol while consuming NADPH(Balestri et al., 2022; Singh et al., 2021) Because NADPH is required for the regeneration of reduced glutathione, excessive polyol-pathway activity weakens endogenous antioxidant defenses and increases cellular susceptibility to oxidative stress. Sorbitol is subsequently converted into fructose by sorbitol dehydrogenase, accompanied by the reduction of NAD^+^ to NADH (Jannapureddy et al., 2021). The resulting increase in the NADH/NAD^+^ ratio produces a pseudohypoxic state even when oxygen is present. Meanwhile, intracellular accumulation of sorbitol and fructose increases osmotic pressure, promotes advanced glycation-product formation, and aggravates mitochondrial ROS production.

These metabolic disturbances are particularly harmful to peripheral nerves and vascular endothelial cells. Osmotic stress, glutathione depletion, pseudohypoxia, and ROS accumulation damage Schwann cells, myelin sheaths, axons, and endothelial cells, while reduced nerve-growth-factor synthesis further impairs axonal maintenance and regeneration (Zhang et al., 2025; Nashtahosseini et al., 2025). Neural injury causes loss of protective sensation and increases the risk of unnoticed repetitive trauma, whereas endothelial injury and impaired vasodilation reduce local perfusion and oxygen delivery. Therefore, glucose metabolism disorder promotes DFU progression through the convergence of inflammatory metabolic reprogramming, peripheral neuropathy, vascular dysfunction, and oxidative injury. Together, these processes increase ulcer susceptibility and prevent effective wound healing.

**FIGURE 1:**
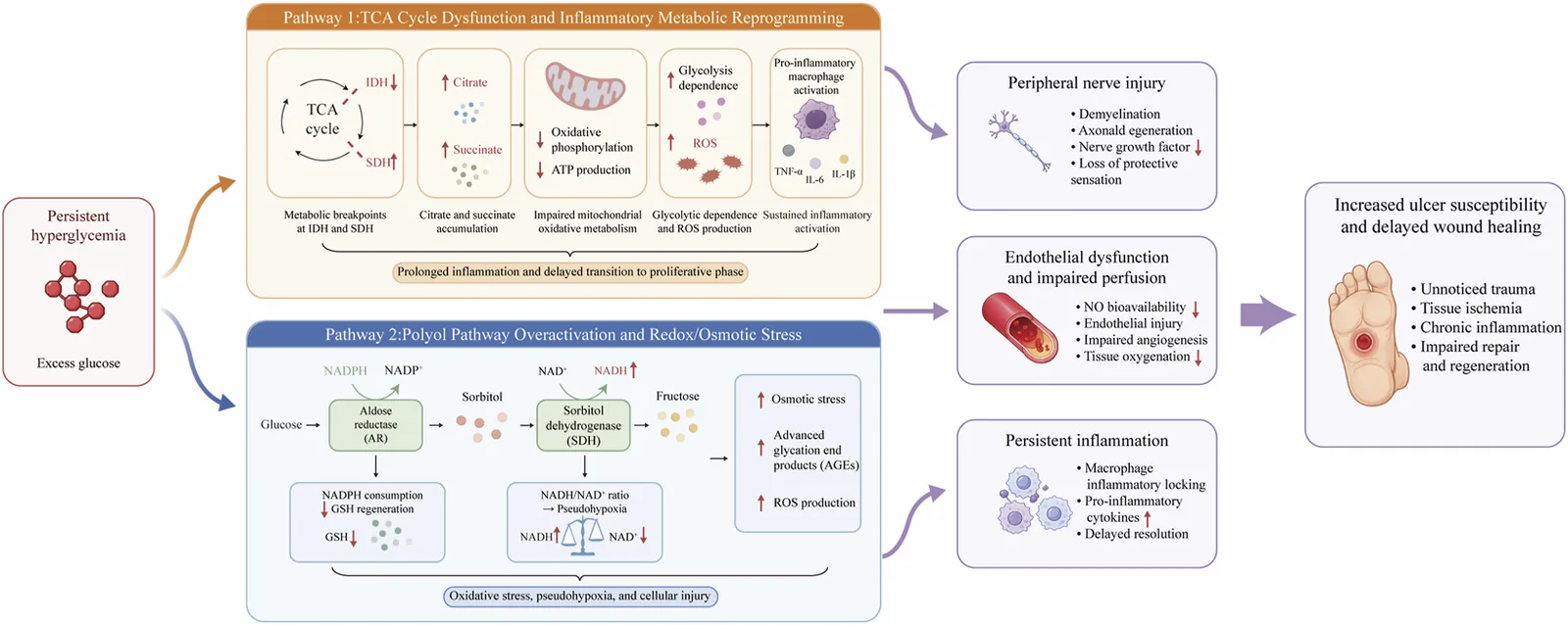
Mechanistic links between glucose metabolism disorders and the progression of diabetic foot ulcers. Persistent hyperglycemia disrupts the TCA cycle and activates the polyol pathway. Metabolic breakpoints at isocitrate dehydrogenase and succinate dehydrogenase promote citrate and succinate accumulation, mitochondrial dysfunction, glycolytic dependence, ROS production, and sustained pro-inflammatory macrophage activation. In parallel, aldose-reductase-mediated glucose-to-sorbitol conversion consumes NADPH and reduces glutathione regeneration, whereas sorbitol-dehydrogenase-mediated fructose production increases the NADH/NAD^+^ ratio. These changes induce osmotic stress, pseudohypoxia, advanced glycation, and oxidative injury. The resulting peripheral nerve injury, endothelial dysfunction, impaired tissue perfusion, and persistent inflammation increase ulcer susceptibility and delay DFU wound healing.

To illustrate this causal sequence more clearly, a schematic representation of the mechanisms linking glucose metabolism disorders to DFU progression is provided in Figure 1.

### 3.2 Disorders of lipid metabolism

Disorders of lipid metabolism often occur secondary to hyperglycemic conditions and disturbances in glucose metabolism, primarily manifesting as hypertriglyceridemia, decreased levels of high-density lipoprotein cholesterol, elevated levels of small, dense low-density lipoprotein, and increased levels of low-carbon saturated lipids (Morze et al., 2022).

Dyslipidemia is a significant contributor to DFU. Chronic hyperlipidemia not only activates the polyol pathway but also induces the production of AGEs and the activation of protein kinase C, leading to increased production of inflammatory factors and growth factors, as well as reduced production of the vasodilator nitric oxide, thereby causing vascular damage (Soyoye et al., 2021). Cholesterol and fasting plasma triglyceride levels are associated with the progression of neuropathy in patients with type 2 diabetes, while obesity and systemic dyslipidemia are independently linked to an increased risk of neuropathy (Eid et al., 2019). Cellular studies have shown that high concentrations of the saturated fatty acid palmitate impair mitochondrial transport in sensory neurons and affect mitochondrial energy production (Rumora et al., 2018). Additionally, increased expression of acylcarnitine in Schwann cells under the influence of long-chain fatty acids is also associated with neurotoxicity and insulin resistance (Eid et al., 2019).

In patients with type 2 diabetes, lipid-lowering therapy reduces cardiovascular risk and shows potential benefits for certain microvascular complications (Steinmetz, 2008). Multifactorial intensive intervention, including lipid-lowering treatment, can lower the risk of autonomic neuropathy (Gaede et al., 2003). Supplementation with monounsaturated fatty acids, such as oleic acid-cultured neurons, can prevent palmitate-induced increases in mitochondrial superoxide anions and the associated decline in ATP levels or insufficient energy production through the formation of lipid droplets (Kwon et al., 2014). In a study involving over 18,000 patients with type 2 diabetes, statin-based lipid-lowering therapy significantly reduced the risk of new-onset diabetic neuropathy and microvascular complications (Kang et al., 2019).

### 3.3 Disorders of protein metabolism

In obese diabetic patients, the plasma amino acid profile undergoes alterations, primarily characterized by elevated levels of branched-chain amino acids (BCAAs) and aromatic amino acids, along with reduced glycine levels (Zakaria et al., 2021). These changes are associated with variations in insulin secretion and the development of insulin resistance (Simonson et al., 2020).

Felig et al. (1969) first reported elevated BCAA levels in the circulation of obese patients, suggesting that hyperaminoacidemia is a manifestation of insulin resistance. A prospective study by Wang et al. (2011) similarly indicated that non-diabetic individuals with elevated plasma BCAA levels have a significantly higher likelihood of developing diabetes over the next decade compared to those with lower plasma BCAA levels. However, current research suggests that the correlation between obesity itself and elevated BCAA levels may be stronger than that with metabolic syndrome and diabetes (Bloomgarden, 2018). It is hypothesized that the molecular mechanisms by which BCAAs induce and exacerbate diabetes primarily involve the activation of the mammalian target of rapamycin complex 1 signaling pathway, which affects insulin signal transduction, influences gluconeogenesis and lipogenesis, leading to or aggravating insulin resistance in patients (Zhou et al., 2019), thereby contributing to the formation or impaired healing of DFU. Among aromatic amino acids, phenylalanine has been shown to catalyze the modification of specific lysine residues on the insulin receptor β via phenylalanyl-transfer RNA synthetase, forming phenylalanylation, which impairs insulin signal transduction and induces insulin resistance in experimental mice (Zhou et al., 2022). This further exacerbates abnormal blood glucose levels, increases vascular and neural damage, and elevates the risk of DFU occurrence.

## 4 Integrated mechanistic framework linking metabolic disorders to cell-specific dysfunction in DFU

The effects of metabolic disorders on DFU wound cells should not be interpreted as isolated changes in glucose, lipid, or amino acid metabolism. Instead, these metabolic abnormalities form an interconnected pathological network. Hyperglycemia drives excessive glycolysis, polyol pathway activation, AGE accumulation, oxidative stress, and pseudohypoxia. Lipid dysmetabolism further aggravates mitochondrial injury through lipid peroxide accumulation, impaired fatty acid oxidation, and membrane damage. Amino acid and protein metabolic disorders contribute to insulin resistance, abnormal arginine utilization, insufficient collagen precursor supply, and excessive ECM degradation. These interconnected metabolic abnormalities converge on macrophages, fibroblasts, and endothelial cells, leading to persistent inflammation, insufficient matrix deposition, and impaired angiogenesis during DFU progression.

### 4.1 Macrophage energy metabolism dysfunction

The wound healing process typically involves three overlapping stages: inflammation, proliferation, and remodeling. Macrophages play a critical role in wound healing, ranging from eliminating potential pathogens to mitigating inflammation after pathogen clearance, and are essential for initiating and sustaining tissue remodeling and regeneration. Persistent excessive inflammation caused by macrophage dysfunction is a significant factor contributing to the difficulty in healing DFU (Li et al., 2021). Although the M1/M2 classification is useful for describing the general inflammatory-to-reparative transition of macrophages during wound healing, it should be regarded as a simplified framework rather than a strict binary definition. In DFU wounds, macrophages exist along a dynamic activation continuum and may display mixed, transitional, or tissue-specific phenotypes depending on wound stage, glucose and lipid stress, hypoxia, infection, and extracellular matrix signals.

In the typical hyperglycemic microenvironment of diabetic wounds, macrophages are exposed to combined glucose, lipid, and amino acid metabolic stress. Glucose metabolic disturbance first drives macrophages toward a glycolysis-dominant state (Ye et al., 2022; O'Neill and Artyomov, 2019). TCA cycle breakpoints at isocitrate dehydrogenase and succinate dehydrogenase promote the accumulation of inflammatory metabolites such as citrate and succinate, which enhance ROS production and inflammatory signaling (Ye et al., 2022). As a result, macrophages tend to retain pro-inflammatory metabolic and transcriptional features rather than acquiring timely repair-associated programs (Liechty et al., 2020; Mills et al., 2016).

Lipid metabolic dysfunction further reinforces this inflammatory metabolic locking. In normal repair, M2 macrophages require mitochondrial oxidative phosphorylation and fatty acid oxidation to support long-term reparative functions (O'Neill and Pearce, 2016; Huang et al., 2014). However, in DFU wounds, free fatty acid accumulation, lipid peroxidation, and mitochondrial membrane damage impair fatty acid oxidation and reduce mitochondrial energy output (Boniakowski et al., 2017). This prevents macrophages from acquiring repair-associated metabolic functions and prolongs the inflammatory phase.

Amino acid metabolism also participates in macrophage dysfunction (Clayton et al., 2024). Under persistent inflammatory stimulation, arginine metabolism is preferentially shunted toward the iNOS pathway, generating excessive nitric oxide and nitrosative stress, while the Arg-1–ornithine–proline axis required for collagen precursor generation and tissue remodeling is weakened (Hellmann et al., 2012). Therefore, macrophage dysfunction in DFU is not only a consequence of enhanced glycolysis but also results from the combined failure of mitochondrial lipid utilization and abnormal amino acid metabolism. This integrated metabolic disorder leads to persistent inflammatory cytokine release, excessive immune-cell recruitment, impaired angiogenesis, and insufficient activation of downstream reparative cells (Sun et al., 2024).

### 4.2 Fibroblast energy metabolism dysfunction

During the proliferative and remodeling phases of wound healing, fibroblasts (FBs) produce provisional ECM, form granulation tissue, and differentiate into myofibroblasts to support wound contraction and re-epithelialization (Wang et al., 2024b). In DFU, fibroblast dysfunction can be understood as the result of three interconnected metabolic pressures: impaired glucose utilization, oxidative/lipid metabolic injury, and abnormal amino acid/protein turnover (Ma et al., 2023b).

First, persistent hyperglycemia disrupts fibroblast glucose metabolism (Hehenberger et al., 1998). Excessive glycolytic byproducts such as lactate can inhibit fibroblast proliferation, while impaired mitochondrial pyruvate utilization reduces ATP production and limits the energy supply required for migration, myofibroblast differentiation, and collagen synthesis. The reported downregulation of pyruvate dehydrogenase kinase in diabetic dermal fibroblasts further indicates that glucose-utilization pathways are dysregulated, contributing to insufficient granulation tissue formation and weakened wound contraction.

Second, lipid peroxidation and oxidative stress amplify fibroblast metabolic exhaustion (Ma et al., 2023a). Hyperglycemia-induced ROS and lipid peroxide accumulation damage mitochondrial function and promote a senescent-like fibroblast phenotype, characterized by reduced proliferative capacity, impaired migration, and diminished matrix-producing activity. Thus, lipid metabolic injury does not act independently but aggravates glucose metabolism-associated mitochondrial dysfunction in fibroblasts.

Third, amino acid and protein metabolic abnormalities directly affect ECM remodeling. Collagen synthesis requires adequate amino acid precursors, including proline and glycine, while macrophage arginine metabolism through the Arg-1–ornithine–proline axis provides metabolic support for matrix deposition (Wynn et al., 2011). In DFU wounds, persistent M1 macrophage activation redirects arginine metabolism toward the iNOS pathway and reduces this reparative metabolic support. At the same time, excessive MMP activity accelerates degradation of newly formed ECM proteins. Therefore, fibroblast dysfunction in DFU reflects a dual defect: insufficient ECM synthesis due to impaired energy and amino acid metabolism, and excessive ECM degradation due to abnormal protein turnover (Ma et al., 2023b; Wan et al., 2021).

### 4.3 Endothelial cell energy metabolism dysfunction

Endothelial cells are essential for neovascularization and restoration of blood supply during wound repair. In DFU, endothelial-cell dysfunction is driven by the convergence of glucose toxicity, lipid metabolic injury, and amino acid metabolism-associated insulin resistance.

Glucose metabolism disorder directly impairs endothelial signaling. Under physiological conditions, insulin activates the PI3K/Akt pathway and promotes endothelial nitric oxide synthase phosphorylation, thereby maintaining nitric oxide production and vascular relaxation (Potenza et al., 2009). In DFU wounds, insulin signaling is weakened, while hyperglycemia increases ROS generation and accelerates nitric oxide inactivation. This reduces nitric oxide bioavailability, weakens vasodilation, and impairs endothelial migration and tube formation. In addition, hyperglycemia-induced PKC-δ activation can upregulate cholesterol metabolism-related enzymes such as HMGCS1 and HMGCR, promoting endothelial apoptosis and further compromising wound revascularization (Qin et al., 2024).

Lipid metabolic disturbance further aggravates endothelial injury. Lipid peroxidation damages cellular membranes and mitochondria, while lipid peroxide accumulation can trigger ferroptosis-like endothelial cell death (Stockwell et al., 2017). This mechanism links systemic dyslipidemia and local oxidative stress to defective angiogenesis in DFU(Okonkwo and DiPietro, 2017). Therefore, endothelial dysfunction is not only a consequence of impaired glucose metabolism but also a manifestation of lipid-peroxidation-driven metabolic cell injury (Rohlenova et al., 2018).

Amino acid metabolism contributes to endothelial dysfunction mainly through systemic and local insulin-resistance mechanisms. Elevated BCAAs and aromatic amino acids can impair insulin signal transduction, thereby indirectly weakening insulin-dependent endothelial nitric oxide production (De Bandt et al., 2023). Meanwhile, inflammatory arginine consumption through the iNOS pathway may disturb the balance between pathological nitric oxide overproduction in inflammatory cells and physiological nitric oxide generation in endothelial cells (Jude et al., 1999). Together, these metabolic alterations produce a vascular microenvironment characterized by reduced nitric oxide bioavailability, oxidative stress, endothelial apoptosis, and insufficient angiogenesis, ultimately delaying DFU wound repair (Okonkwo and DiPietro, 2017).

Taken together, macrophages, fibroblasts, and endothelial cells do not respond to metabolic disorders independently. Instead, they form a metabolically coupled wound-healing network. In the early inflammatory phase, hyperglycemia-driven glycolysis, succinate accumulation, impaired fatty acid oxidation, and abnormal arginine metabolism lock macrophages in a pro-inflammatory state. These macrophages release inflammatory cytokines, ROS, nitric oxide, and MMPs, which further increase oxidative stress and protein degradation in the wound bed. During the proliferative phase, fibroblasts exposed to this environment show mitochondrial dysfunction, reduced collagen synthesis, and impaired myofibroblast differentiation. In parallel, endothelial cells undergo nitric oxide imbalance, oxidative injury, lipid peroxidation, and ferroptosis-like damage, leading to insufficient angiogenesis. Therefore, DFU progression can be conceptualized as a transition from systemic substrate metabolic disorder to local multicellular metabolic failure: glucose metabolism mainly initiates hyperglycemic stress and glycolytic reprogramming, lipid metabolism amplifies mitochondrial and membrane injury, and amino acid/protein metabolism determines inflammatory resolution and ECM remodeling capacity.

**FIGURE 2:**
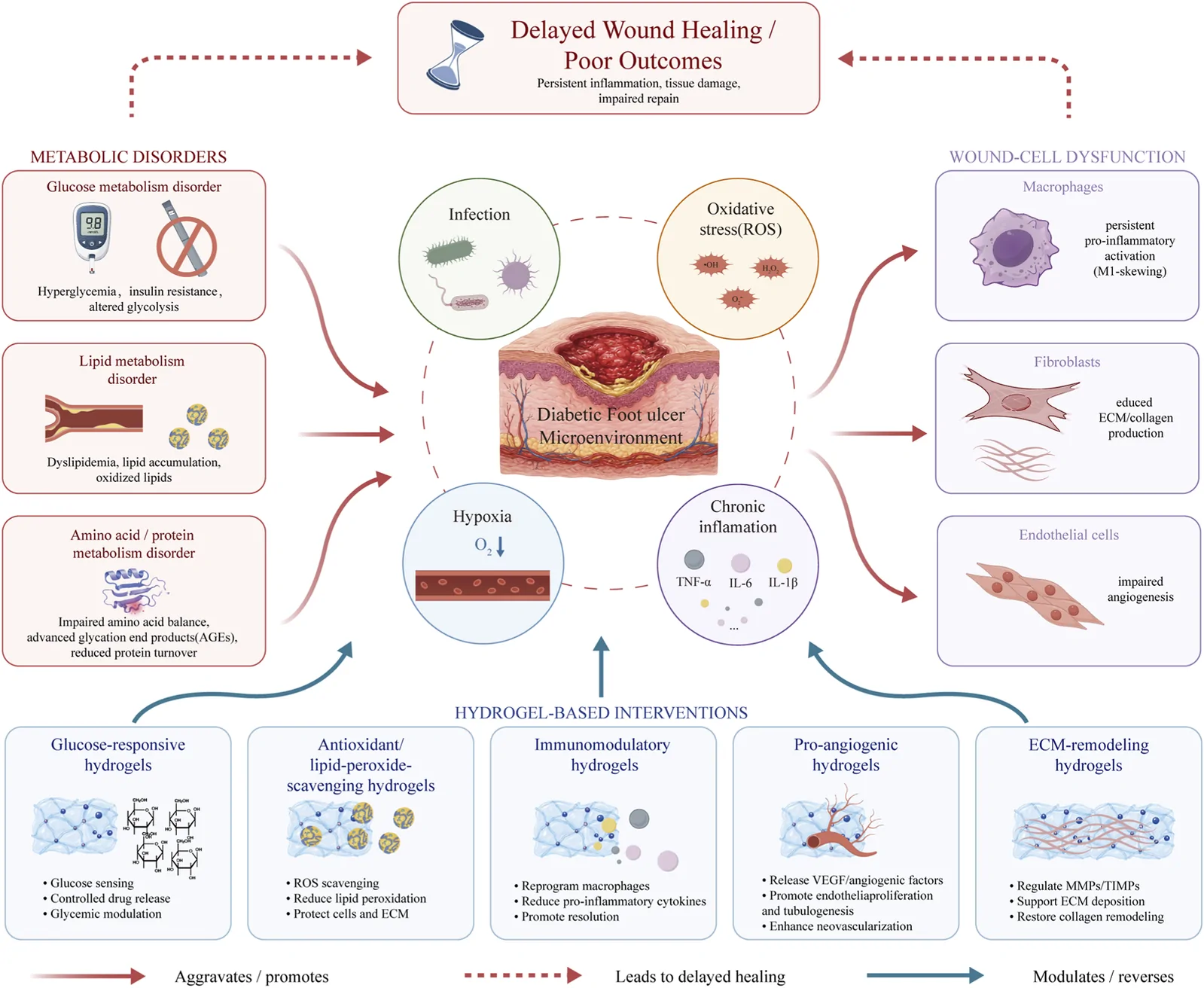
Conceptual framework linking metabolic disorders, wound-cell dysfunction, and hydrogel-based interventions in diabetic foot ulcers. Glucose, lipid, and amino acid/protein metabolism disorders jointly contribute to the pathological DFU microenvironment by aggravating infection, oxidative stress, hypoxia, and chronic inflammation. These abnormalities impair the functions of key wound-healing cells, including macrophages, fibroblasts, and endothelial cells, resulting in persistent pro-inflammatory activation, reduced ECM/collagen production, impaired angiogenesis, and delayed wound healing. Hydrogel-based interventions may modulate this pathological network through glucose-responsive delivery, antioxidant and lipid-peroxide-scavenging activity, immunomodulation, pro-angiogenic regulation, and ECM remodeling, thereby restoring tissue homeostasis and promoting wound repair.

To further integrate the above mechanisms, we constructed an original conceptual framework summarizing the interactions among metabolic disorders, pathological wound microenvironment, wound-cell dysfunction, and hydrogel-based therapeutic interventions (Figure 2). In this framework, glucose, lipid, and amino acid/protein metabolic disorders jointly aggravate infection, oxidative stress, hypoxia, and chronic inflammation in the DFU microenvironment. These pathological factors further impair macrophage, fibroblast, and endothelial-cell functions, resulting in persistent inflammation, defective ECM remodeling, impaired angiogenesis, and delayed wound healing. Hydrogel-based delivery systems may intervene in this vicious cycle by regulating glucose metabolism, scavenging ROS and lipid peroxides, modulating immune responses, promoting angiogenesis, and supporting ECM remodeling.

## 5 The advantages of hydrogels as a targeted delivery system for DFU

Hydrogels can establish a favorable environment by maintaining appropriate temperature, humidity, and pH levels, thereby promoting the processes of granulation tissue formation and re-epithelialization (Lima and Passos, 2021). Additionally, they exhibit certain antibacterial and anti-inflammatory properties, which help reduce the risk of bacterial colonization during wound healing (Hu and Xu, 2020). With advancements in materials science, various natural and synthetic biomaterials have been used to synthesize hydrogels, including alginate, collagen, hyaluronic acid, and chitosan. These materials provide effective cell affinity and biocompatibility. Wang et al. (2023) developed a natural carrier-free binary small-molecule hydrogel composed of baicalin and arnica alkaloids. These compounds can self-assemble into carrier-free small-molecule hydrogels through non-covalent interactions, including electrostatic attraction, π–π stacking, and hydrogen bonding. This binary small-molecule hydrogel exhibits excellent extensibility and injectability, making it suitable for various types of wounds. Furthermore, the synergistic effects of baicalin and arnica alkaloids have been shown to modulate inflammatory factors and inhibit bacterial virulence factors, thereby promoting the wound healing process. Liu et al. (2020) developed a self-assembling hydrogel that can be fully absorbed by the body without local or systemic toxicity, accelerating the regeneration of skin wounds infected with methicillin-resistant *Staphylococcus aureus* (MRSA). Hyaluronic acid hydrogels have also been applied in the treatment of DFU. Xu et al. (2022) modified methacrylated hyaluronic acid with phenylboronic acid to create a composite with a three-dimensional network structure similar to skin tissue. When applied to chronic diabetic wounds, it effectively promotes angiogenesis (enhancing vascular endothelial growth factor and CD31 expression) and reduces inflammation (lowering IL-6 levels and increasing IL-10 levels), thereby facilitating rapid repair of DFU wounds.

From the perspective of biomaterial design, advanced wound-healing hydrogels should not only provide passive coverage but also integrate tunable network structures, appropriate porosity, mechanical adaptability, bioactive molecule loading, and controlled release profiles. For example, Islam et al. (2024) developed silk-enriched PVA hydrogels containing ROS-scavenging dendrimers, in which silk fibroin provided structural support, dendrimers contributed antioxidant activity, and the PVA network served as the hydrogel matrix. This study illustrates how polymer composition and functional antioxidant components can be rationally combined to regulate the oxidative microenvironment of DFU wounds.

From the viewpoint of delivery-system engineering, DFU hydrogels may be further integrated with surface-mediated assembly strategies, nanoscale carriers, or mineralized delivery modules to improve the spatial retention and controlled release of nucleic acids, proteins, exosomes, and metabolic regulators. For example, DNA–amorphous calcium phosphate nanocomposite spheres have been developed for surface-mediated gene delivery, suggesting that mineral-based superficial assembly may provide a useful strategy for coupling gene delivery modules with hydrogel wound dressings (Oyane et al., 2016). Such strategies may be particularly valuable for delivering metabolic regulatory nucleic acids or gene-editing components in chronic diabetic wounds. Hydrogels play multifaceted roles in DFU treatment, and their diversity and designability make them promising for managing complex wounds in DFU.

In the following sections, metabolism-targeted hydrogel delivery systems are discussed in a more restricted mechanistic sense. Specifically, direct metabolism-targeted systems refer to hydrogels that regulate metabolic substrates, metabolic enzymes, metabolic pathways, or metabolite-driven cellular dysfunction, such as glucose sensing or consumption, modulation of glycolysis, fatty acid oxidation, amino acid metabolism, mitochondrial function, or ferroptosis. In contrast, hydrogels with antibacterial, antioxidant, anti-inflammatory, or pro-angiogenic activities are considered metabolism-associated microenvironmental regulators only when their effects can be mechanistically linked to hyperglycemia-induced oxidative stress, lipid peroxidation, hypoxia, mitochondrial dysfunction, or impaired cellular energy metabolism. To facilitate comparison across different hydrogel systems, a critical summary of hydrogel composition, delivered cargo, targeted metabolic pathway, target cell type, experimental model, therapeutic outcome, mechanistic evidence, and major limitations is provided in Table 1.

**TABLE 1:** Critical comparison of representative hydrogel delivery systems targeting metabolic dysfunction in DFU.

Hydrogel system	Hydrogel composition	Delivered cargo	Targeted metabolic pathway/process	Target cell type	Experimental model	Therapeutic outcome	Mechanistic evidence	Major limitation	References
TP-Mg@PSG	PVA/SA/gelatin hydrogel	TP-MgNPs	Hyperglycemia-related ROS/infection stress	Bacteria, inflammatory cells	MRSA-infected diabetic wound	Antibacterial, anti-inflammatory, pro-healing	ROS scavenging, MMP-9/VEGF regulation	Indirect metabolic regulation	Hu et al. (2024)
Dex-GMA/ConA/chitosan hydrogel	Dex-GMA/ConA microspheres in chitosan	Insulin	Glucose sensing and insulin release	Glucose-responsive system	Glucose-responsive release model	Sustained glucose-dependent insulin release	Glucose–ConA competitive binding	Limited DFU wound evidence; ConA biosafety	Yin et al. (2019)
GOX/CAT hybrid hydrogel	Metal-polyphenol hydrogel	GOX, CAT	Glucose consumption and oxygen generation	Macrophages, endothelial cells	MRSA-infected diabetic wound	Reduced hyperglycemia/hypoxia, promoted healing	GOX consumes glucose; CAT produces O_2_	Enzyme stability and H_2_O_2_ control	Gong et al. (2025)
Silk/PVA antioxidant hydrogel	Silk fibroin/PVA hydrogel	ROS-scavenging dendrimers	Oxidative metabolic stress	Wound-resident cells	Diabetic wound	ROS reduction and wound repair	Dendrimer-mediated ROS scavenging	Indirect metabolic regulation	Islam et al. (2024)
Organoselenium hydrogel	Tissue-conformable organoselenium hydrogel	Organoselenium groups	Lipid peroxidation/AGE-RAGE	Wound inflammatory cells	Diabetic wound	Reduced LPOs and accelerated repair	GPx-like LPO clearance	Long-term selenium safety	Zhao et al. (2026a)
MXene/PPy/ApoV hydrogel	MXene/PPy conductive hydrogel	ApoVs	AMPK-mediated FAO	Macrophages	Infected diabetic wound	M2 polarization and wound repair	AMPK activation, FAO recovery	Nanofiller safety; ApoV standardization	Yan et al. (2026)
AP@HA-SiInj Gel	HA-based injectable hydrogel	Arginine, bioactive molecules	Arginine metabolism	Macrophages, fibroblasts	Diabetic wound	M2 polarization, collagen deposition	Arg-1-related amino acid metabolism	Dose and iNOS/Arg-1 balance	Wan et al. (2025)
ADSC-exo@MMP-PEG hydrogel	MMP-responsive PEG hydrogel	ADSC exosomes	Abnormal protein turnover	ECM-remodeling cells	Diabetic wound with high MMPs	ECM protection and repair	MMP-triggered gel degradation and exosome release	Indirect protein-metabolism regulation	Jiang et al. (2022)
4-OI hydrogel	4-OI-loaded exosome/GelMA hydrogel	4-OI	Glycolysis/Nrf2 pathway	Macrophages	Diabetic wound	M2 polarization, angiogenesis, granulation	GAPDH inhibition, Nrf2 activation	Dose window and off-target effects	Shen et al. (2024)
DMM/GelMA hydrogel	Injectable GelMA hydrogel	DMM	SDH–succinate–ROS axis	Macrophages	Diabetic wound	Reduced inflammation, promoted angiogenesis	SDH inhibition and ROS reduction	Long-term SDH inhibition safety	Shao et al. (2025)
GPP@ZnBG hydrogel	GOx/CAT ion-releasing hydrogel	GOx, CAT, Zn/Ca/Si ions	Glucose/ROS/pH regulation	Fibroblasts	Diabetic wound; scRNA-seq	Collagen deposition and granulation	GOx/CAT cascade, ion-mediated signaling	Enzyme and ion-release stability	Zhao et al. (2026b)
AFGKLT hydrogel	Anisotropic nanofiber hydrogel	None	Metabolism-supportive structure	Fibroblasts	Diabetic wound	Directed migration and wound contraction	ECM-like physical guidance	Lacks direct metabolic evidence	Kim et al. (2025)
HFN-RGE hydrogel	HA/F127 thermoelectric hydrogel	Ginseng-derived exosomes	Endothelial dysfunction under metabolic stress	Endothelial cells	Ischemic/diabetic wound	Angiogenesis and tube formation	Electric-field and exosome signaling	Mainly pro-angiogenic, not direct metabolic targeting	Tan et al. (2025)
MMP-9-responsive anti-ferroptosis hydrogel	MMP-9-responsive hydrogel	Anti-ferroptosis agents/nanozymes	Lipid-peroxidation-driven ferroptosis	Endothelial cells	Diabetic wound with high MMP-9	Reduced ferroptosis, improved angiogenesis	MMP-9-triggered release; ferroptosis marker reduction	Needs stronger metabolic flux evidence	Lin et al. (2025)

### 5.1 Hydrogel delivery system targeting glucose metabolism disorders

**FIGURE 3:**
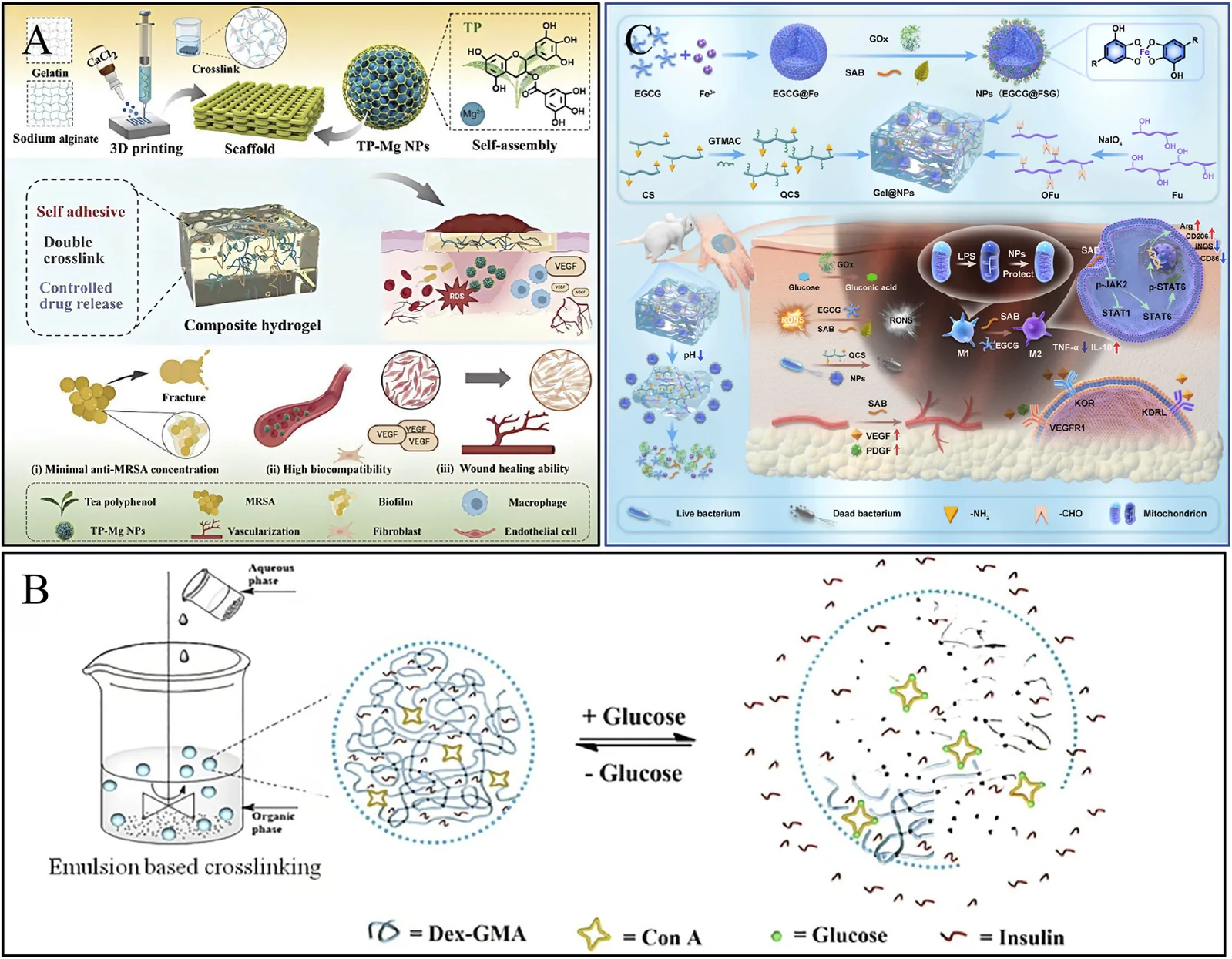
The Construction Process and Structure of Representative Hydrogels Involved in Glucose-Metabolism Regulation and Hyperglycemia-Associated Microenvironment Modulation **(A)** Preparation of TP-Mg@PSG Double-Network Hydrogel and Its Promotion of DFU Healing Infected with MRSA, Along with Its Mechanism of Action. Reproduced from ref (Hu et al., 2024). Copyright 2024 Wiley. **(B)** Fabrication process and glucose-responsive structural change of microspheres. Reproduced from ref (Yin et al., 2019). Copyright 2018 Elsevier. **(C)** Design strategy diagram of a hydrogel dressing containing EGCG@FSG for the treatment of DFU. Reproduced from ref (Gong et al., 2025). Copyright 2025 Elsevier.

Impaired glucose metabolism in diabetic foot patients leads to MRSA biofilm infections, posing a significant obstacle to the healing process and carrying a high risk of life-threatening complications. Due to microvascular occlusion in diabetic foot and the tenacity of MRSA biofilms, controlling and effectively treating MRSA infections remains challenging. To address this, Hu et al. (2024) developed a polyvinyl alcohol/sodium alginate/gelatin (PSG) double-network hydrogel loaded with novel tea polyphenol self-assembled magnesium nanoparticles (TP-MgNPs), designated as TP-Mg@PSG (Figure 3A). In the acidic microenvironment characteristic of MRSA-infected wounds, this hydrogel degrades and effectively releases TP-MgNPs. The released nanoparticles can penetrate and efficiently eradicate stubborn MRSA biofilms in a dose-dependent manner by disrupting bacterial DNA and inducing its degradation. Simultaneously, leveraging the potent antioxidant and free radical scavenging capabilities of tea polyphenols, this delivery system effectively scavenges reactive oxygen species (ROS) *in vitro* and modulates the local inflammatory microenvironment of the wound by downregulating the expression levels of matrix metalloproteinase-9 (MMP-9) and interleukin-10 (IL-10). Furthermore, the system significantly upregulates the expression of vascular endothelial growth factor (VEGF), promoting angiogenesis in the wound. Therefore, TP-Mg@PSG should not be interpreted as a direct glucose-metabolism-targeted hydrogel because it does not directly sense or consume glucose. Instead, its relevance to glucose-metabolism disorder lies in its ability to alleviate downstream pathological consequences of the hyperglycemic wound microenvironment, including ROS accumulation, infection-driven metabolic stress, and inflammation-associated impairment of tissue repair. Thus, this system is better classified as an indirect metabolism-associated microenvironmental regulator.

Although effective clearance of infection and modulation of the local microenvironment are crucial for early wound intervention, fundamentally breaking the persistent vicious cycle of “infection-poor healing” in diabetic foot requires direct correction of local glucose metabolism disorders. In constructing an intelligent hydrogel delivery system targeting glucose metabolism, concanavalin A (ConA) has emerged as a core molecule due to its exceptional glucose concentration-sensing and metabolic regulation potential. ConA is a plant lectin with multivalent binding sites that can reversibly and competitively bind to glucose and glucose-containing polymers (such as dextran) (Shen et al., 2020). When glucose concentrations in the wound site or blood abnormally increase, free glucose molecules competitively occupy the binding sites on ConA, leading to the dissociation or swelling of the originally tightly cross-linked polymer network, thereby triggering the intelligent release of encapsulated metabolic regulatory drugs (such as insulin). Conversely, when glucose metabolism returns to balance and glucose concentrations decrease, the release network re-closes (Wu et al., 2011). Based on this sophisticated glucose metabolism-sensing and regulatory mechanism of ConA, Yin et al. (2019) ingeniously developed a “synthetic artificial pancreas”-type composite hydrogel delivery system to achieve long-term, closed-loop management of glucose metabolism. The research team first employed a high-shear emulsification cross-linking method to precisely encapsulate insulin within glucose-responsive microspheres composed of glycidyl methacrylate-modified dextran (Dex-GMA) and ConA. These microspheres were then embedded into a chitosan hydrogel scaffold with excellent biocompatibility (Figure 3B). This bilayer delivery system not only significantly reduced the risk of drug burst release commonly associated with free microspheres but also provided long-term physical protection for the microspheres through the sustained degradation of the chitosan hydrogel. More importantly, the released microspheres perfectly retained the glucose-responsive properties of the ConA system: over a 12-day release period, when exposed to high glucose levels simulating diabetic pathology, ConA within the system rapidly sensed the change and released insulin with full biological activity to intervene in glucose metabolism abnormalities. Conversely, under basal blood glucose concentrations, the insulin release rate automatically decreased. This hydrogel system, which integrates sustained drug supply with “closed-loop” intelligent regulation of glucose metabolism, offers a novel and clinically translatable approach for long-term correction of metabolic disorders in the microenvironment of DFU. This system represents a direct *metabolism*-targeted hydrogel because the release behavior is governed by local glucose concentration, and the therapeutic payload, insulin, directly corrects glucose metabolic imbalance rather than only suppressing secondary inflammation or infection.

However, beyond the passive sensing and regulation of blood glucose concentration, actively consuming excess glucose at the wound site through catalytic means to reduce the “substrate” of metabolic disorders at the source represents another efficient pathway for reconstructing the metabolic microenvironment. In this strategy, glucose oxidase (GOX) plays a central role. GOX is a highly specific biocatalytic enzyme that catalyzes the reaction between glucose and oxygen to produce gluconic acid and hydrogen peroxide (H_2_O_2_). In diabetic wounds, GOX not only directly reduces local hyperglycemia through the “glucose scavenging” effect, cutting off the energy source for bacterial colonization and chronic inflammation, but also acts as an initiator to trigger subsequent cascade reactions (Qi et al., 2026). Based on this concept of cascade catalytic reconstruction of metabolism, Gong et al. (2025) recently developed an integrated GOX-based metal-polyphenol nanocomposite hybrid hydrogel platform (Figure 3C). This system ingeniously co-encapsulates GOX with catalase (CAT) and metal-polyphenol nanoparticles. Its core logic lies in utilizing the efficient catalytic capability of GOX to convert excess glucose at the wound site into H_2_O_2_; subsequently, CAT or metal components with nanozyme activity in the system further decompose the generated H_2_O_2_, consuming glucose while producing oxygen (O_2_), thereby alleviating the common hypoxic metabolic dilemma in diabetic wounds. Experimental results demonstrate that this GOX-mediated cascade reaction not only achieves a metabolic phenotypic shift in the wound from “hyperglycemic and hypoxic” to “normal glucose levels and oxygen-rich,” but also significantly reduces oxidative stress induced by hyperglycemia. This “metabolic reprogramming” effect synergistically reshapes the immune microenvironment, inducing macrophage polarization from pro-inflammatory M1 to pro-repair M2 phenotypes. The antibacterial and pro-angiogenic effects observed in this system should be regarded as downstream therapeutic outcomes of metabolic microenvironment remodeling, rather than the primary basis for defining it as *metabolism*-targeted. Its direct metabolic mechanism is the GOX/CAT-mediated regulation of two key metabolic variables in DFU wounds: excessive glucose and insufficient oxygen. Compared to traditional passive drug delivery systems, this “multifunctional metabolic regulation platform” based on GOX catalytic reconstruction provides a more proactive and systematic biomaterial solution for addressing the challenging healing of DFU by actively intervening in the hub of energy metabolism.

These examples indicate that glucose-related hydrogel systems should be distinguished according to whether they directly regulate glucose metabolism or indirectly alleviate hyperglycemia-associated microenvironmental stress.

In summary, hydrogel systems related to glucose metabolic disorders can be divided into direct glucose-metabolism-targeted systems and indirect metabolism-associated microenvironmental regulators. ConA-based hydrogels and GOX/CAT-based catalytic hydrogels directly regulate glucose metabolism by glucose-responsive insulin release or local glucose consumption, respectively. In contrast, TP-Mg@PSG mainly mitigates hyperglycemia-associated downstream stress, including ROS accumulation, infection-related metabolic burden, and inflammatory amplification. Therefore, glucose-related hydrogel strategies should be evaluated not only by wound closure or antibacterial outcomes but also by whether they directly reduce glucose burden, improve oxygen availability, or provide glucose-dependent therapeutic release. This distinction is important for avoiding overinterpretation of general microenvironmental improvement as direct metabolic targeting.

### 5.2 Hydrogel delivery system targeting lipid metabolism disorders

Lipid dysmetabolism also plays an extremely critical role in the mechanism of impaired wound healing. The intense oxidative stress induced by a high-glucose environment leads to severe lipid peroxidation, damaging the integrity of mitochondrial membranes (Volpe et al., 2018). Meanwhile, abnormal deposition of free fatty acids in the wound inhibits fatty acid oxidation (FAO) within macrophage mitochondria, cutting off the core energy source required for their transition to the M2 repair phenotype (Huang et al., 2014; Louiselle et al., 2021).

**FIGURE 4:**
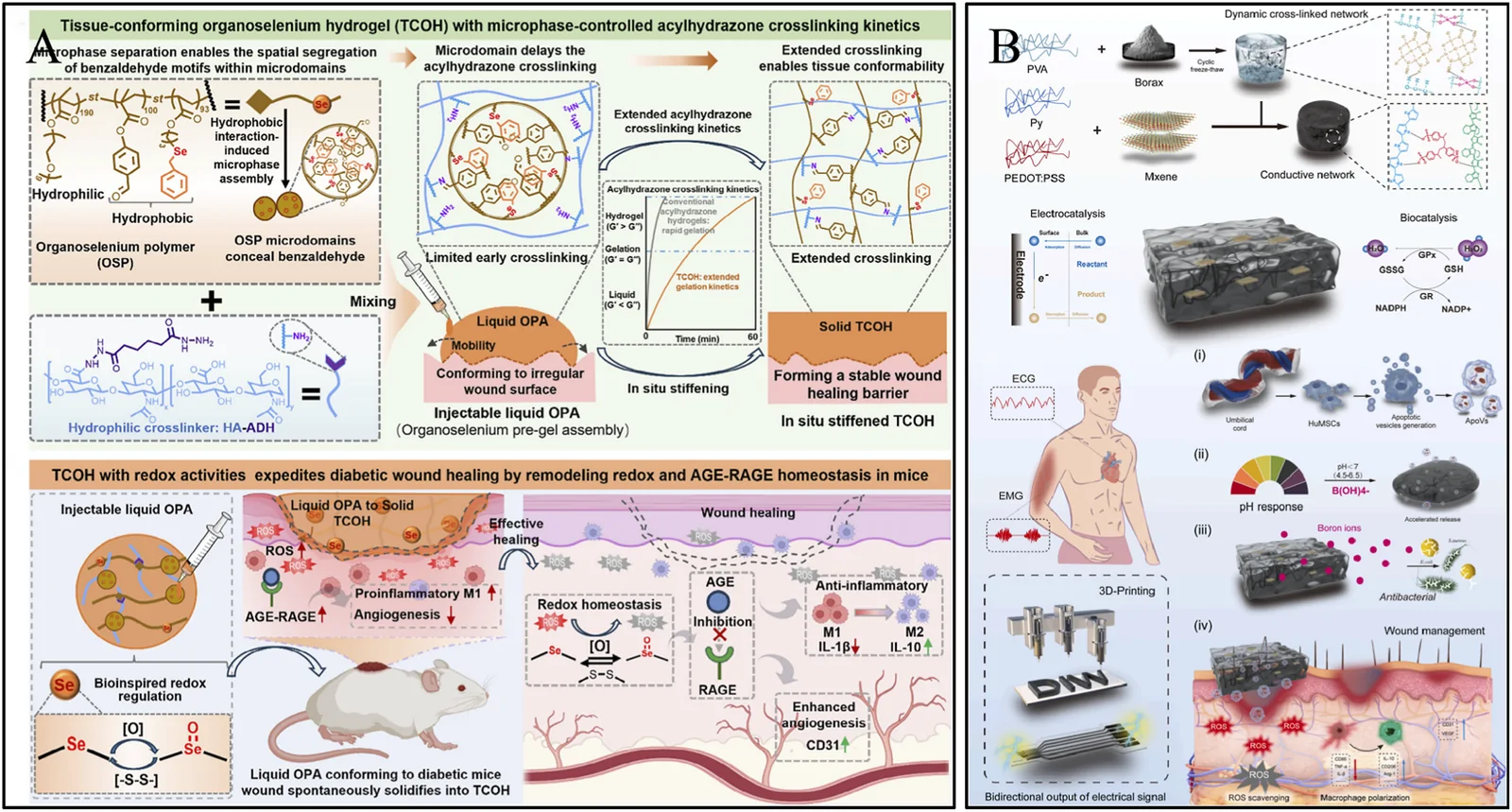
The Construction Process and Structure of Representative Hydrogels Targeting Lipid Metabolism Disorders. **(A)** Schematic illustration of the tissue-conforming organoselenium hydrogel (TCOH) for DFU healing. Reproduced from Zhao et al. (2026a). Copyright 2026 Wiley. **(B)** Schematic diagram of the overall study design. Reproduced from Yan et al. (2026). Copyright 2026 Elsevier.

Given the profound crosstalk between glucose and lipid metabolism at the mitochondrial level, emerging hydrogel delivery systems are gradually evolving from simple correction of glucose metabolism toward deeper “lipid metabolism reprogramming.” Zhao et al. (2026a) recently developed a tissue-conformable organoselenium composite hydrogel, which achieves physical interlocking with wound tissue through microphase-controlled hydrazide crosslinking kinetics (Figure 4A). The core component, organoselenium, exhibits glutathione peroxidase (GPx)-like activity, enabling targeted clearance of harmful lipid peroxides (LPOs) induced by hyperglycemia in the wound. By remodeling the local lipid microenvironment, this effectively blocks the cascade activation of the AGE-RAGE signaling pathway and significantly accelerates tissue repair in DFU. Therefore, the metabolic target of this hydrogel is not general oxidative stress, but lipid-peroxide accumulation and lipid-peroxidation-driven AGE-RAGE activation under hyperglycemic conditions.

Meanwhile, significant progress has been made in intervening in the immunometabolic phenotype of macrophages through intelligent carriers. Yan et al. (2026) reported a conductive double-network hydrogel based on Ti3C2Tx MXene and polypyrrole (PPy). This system exhibits sensitive pH-responsive properties, enabling the on-demand release of loaded apoptotic vesicles (ApoVs) in the infected microenvironment of DFU (Figure 4B). The conductive system not only utilizes the nanozyme activity of MXene to synergistically scavenge oxidative stress but, more importantly, the released ApoVs act as efficient metabolic modulators. They activate key energy metabolism hub pathways such as AMPK *in situ*, thereby forcibly restarting the impaired mitochondrial fatty acid oxidation (FAO) process in macrophages. This “dual metabolic reprogramming” strategy—on one hand, alleviating cellular metabolic burden by clearing lipotoxic products (e.g., LPOs and AGEs), and on the other hand, enhancing cellular energy output by activating FAO—shifts macrophages from a predominantly pro-inflammatory metabolic state toward repair-associated functional programs through energy substrate switching (Figure 3B). Thus, the rationale for including this system is its ability to restore macrophage lipid catabolism through AMPK-mediated FAO activation, rather than its anti-inflammatory outcome alone. When conductive or carbon-based nanofillers are incorporated into hydrogel matrices, their dispersion stability, surface functionalization, and cytocompatibility should be carefully considered (Chen et al., 2021).

In summary, targeting lipid homeostasis through metabolic reprogramming has emerged as one of the key strategies to overcome the bottleneck in diabetic foot ulcer healing. From passively clearing lipid peroxides to alleviate microenvironmental lipotoxicity to actively activating mitochondrial FAO to remodel the energy substrates of macrophages, such intelligent delivery systems based on advanced hydrogels profoundly illustrate the core value of “dual metabolic reprogramming” in reversing immune imbalance in wounds. This not only enriches our understanding of the mechanisms underlying the refractory healing of complex chronic wounds but also signifies a shift in the intervention targets of biomaterials—from superficial regulation of cellular phenotypes toward deeper modulation of mitochondrial energy metabolism. In the future, the design of next-generation intelligent dressings for DFU should further focus on the dynamic network of glucose-lipid metabolic crosstalk, aiming to develop multimodal composite platforms capable of spatiotemporally precise regulation of multiple metabolic hubs (such as the AMPK cascade and the AGE-RAGE pathway). This intervention model, which comprehensively reshapes the immune microenvironment from the underlying energy metabolic network. These lipid metabolism-related hydrogel systems illustrate two complementary strategies: reducing lipid-peroxidation-derived injury and restoring mitochondrial lipid utilization. Compared with general antioxidant hydrogels, organoselenium hydrogels have a clearer lipid-metabolic rationale because they target LPO accumulation and AGE-RAGE activation. Similarly, ApoV-loaded conductive hydrogels are not merely anti-inflammatory systems; their major value lies in AMPK-mediated FAO restoration and macrophage immunometabolic remodeling. Nevertheless, long-term nanomaterial biosafety, ApoV standardization, and direct metabolic flux evidence remain important challenges before these systems can be translated clinically.

### 5.3 Hydrogel delivery systems targeting amino acid metabolism and abnormal protein turnover

Beyond the profound crosstalk in glucose and lipid metabolism, severe imbalances in protein and amino acid metabolism also serve as a core driver behind the “prolonged non-healing” of diabetic foot wounds. In normal tissue repair, macrophages metabolize L-arginine via the arginase-1 (Arg-1) pathway to produce ornithine and proline, thereby providing crucial amino acid precursors for fibroblast collagen synthesis (Wang et al., 2025). However, in the hyperglycemic and highly inflammatory microenvironment of DFU, M1 macrophages dominate and shunt large amounts of arginine into the iNOS pathway, generating excessive nitric oxide (NO). This not only exacerbates local nitrosative stress but also completely cuts off the supply of raw materials for ECM protein synthesis (Szondi et al., 2021; Boniakowski et al., 2017). Concurrently, matrix metalloproteinases (MMPs) in the wound bed exhibit pathologically sustained overexpression, leading to excessive degradation of newly formed ECM proteins (such as collagen and elastin) (Fu et al., 2022). This dual collapse in protein metabolism—characterized by “depletion of synthetic precursors” and “excessive degradation”—prevents the wound from establishing a stable tissue remodeling scaffold.

**FIGURE 5:**
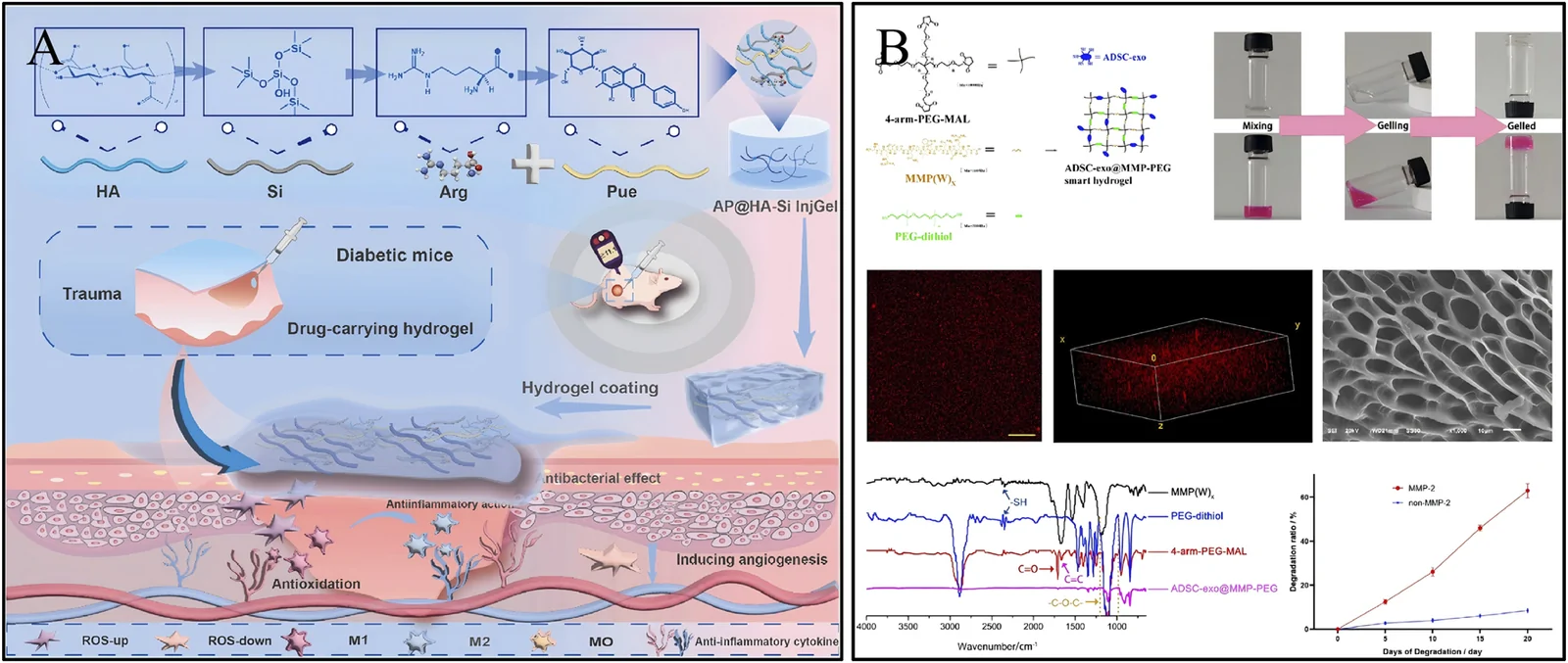
The Construction Process and Structure of Representative Hydrogels Targeting Amino Acid Metabolism and Abnormal Protein Turnovers. **(A)** Schematic illustration of the design, synthesis, and therapeutic mechanisms of AP@HA–Si InjGel for diabetic wound healing. Reproduced from Wan et al. (2025). Copyright 2025 KeAi. **(B)** Preparation and characterization of ADSC-exo@MMP-PEG smart hydrogel. Reproduced from Jiang et al. (2022). Copyright 2022 Elsevier.

To address these protein and amino acid metabolic barriers, emerging hydrogel systems have demonstrated considerable potential through “metabolic intervention” and “microenvironment-responsive” strategies. First, local delivery of specific metabolically regulatory amino acids via hydrogels can reshape cellular protein synthesis pathways at the source. For instance, a recent study (Wan et al., 2025) developed a hyaluronic acid-based composite injectable hydrogel (e.g., AP@HA-SiInj Gel) loaded with arginine and bioactive molecules (Figure 5A). By providing sustained *in situ* release of arginine, this hydrogel directly intervenes in the amino acid homeostasis of local immune cells. The supplementation of exogenous arginine not only restores mitochondrial membrane potential but, more importantly, forcibly redirects the amino acid metabolic trajectory of macrophages, promoting their polarization toward an M2 phenotype. This, in turn, restores the synthesis capacity of collagen precursors and significantly accelerates ECM deposition in wound granulation tissue.

Second, to tackle the problem of hyperactive protein degradation in DFU wounds, researchers (Jiang et al., 2022) have ingeniously transformed a “pathological disadvantage” into a “therapeutic advantage” by developing MMP-responsive smart hydrogels (Figure 5B). Traditional strategies often rely on MMP inhibitors, which may interfere with normal physiological processes. In contrast, recent studies have introduced MMP-sensitive peptides (e.g., polyethylene glycol derivatives containing specific cleavage sites) as crosslinkers into hydrogel networks. For example, in a smart hydrogel loaded with adipose-derived stem cell exosomes (ADSC-exo@MMP-PEG), when the hydrogel encounters abnormally elevated MMPs in the DFU wound, the gel backbone is specifically cleaved and depolymerized. This design offers dual therapeutic effects: the hydrogel skeleton itself acts as a “competitive substrate” that consumes the local excess of proteolytic enzymes, thereby protecting endogenous neovascularization and ECM proteins from degradation. Simultaneously, as the gel degrades, the encapsulated pro-healing exosomes are released on-demand into the deeper wound bed.

This biomaterial strategy does not directly regulate protein synthesis pathways in the same manner as amino acid supplementation. Instead, it targets abnormal protein turnover in DFU by responding to excessive protease activity, reducing ECM degradation, and releasing pro-healing exosomes on demand. Therefore, MMP-responsive hydrogels are better interpreted as systems that regulate metabolism-associated ECM remodeling rather than direct protein-metabolism-targeted hydrogels.

### 5.4 Hydrogel delivery system targeting macrophage metabolic dysfunction

The functional state of macrophages is closely linked to their energy metabolism. Classically, pro-inflammatory macrophage states are associated with enhanced glycolysis, whereas repair-associated macrophage programs often require mitochondrial oxidative phosphorylation (OXPHOS) and fatty acid oxidation to sustain long-term reparative activity (O'Neill and Pearce, 2016). However, the M1/M2 framework should not be interpreted as a strict binary classification (Murray et al., 2014). Macrophages in chronic diabetic wounds exhibit temporal and phenotypic heterogeneity, and their activation states may include mixed or transitional phenotypes rather than two fixed endpoints (Aitcheson et al., 2021). In the hyperglycemic and hypoxic microenvironment of DFU, mitochondrial dysfunction and persistent glycolytic pressure can maintain macrophages in a chronic pro-inflammatory metabolic state (O'Neill and Pearce, 2016; Boniakowski et al., 2017). This metabolic locking not only limits the acquisition of repair-associated functions but also exacerbates wound inflammation through lactate accumulation and excessive ROS(Lu et al., 2025). Therefore, hydrogel-based immunometabolic regulation should be understood as reshaping macrophage metabolic trajectories rather than simply converting M1 macrophages into M2 macrophages.

The examples included in this section are direct immunometabolism-targeted hydrogels because the delivered agents act on defined metabolic enzymes or pathways, including GAPDH-mediated glycolysis and SDH/succinate-dependent mitochondrial ROS production, rather than merely changing M1/M2 marker expression.

**FIGURE 6:**
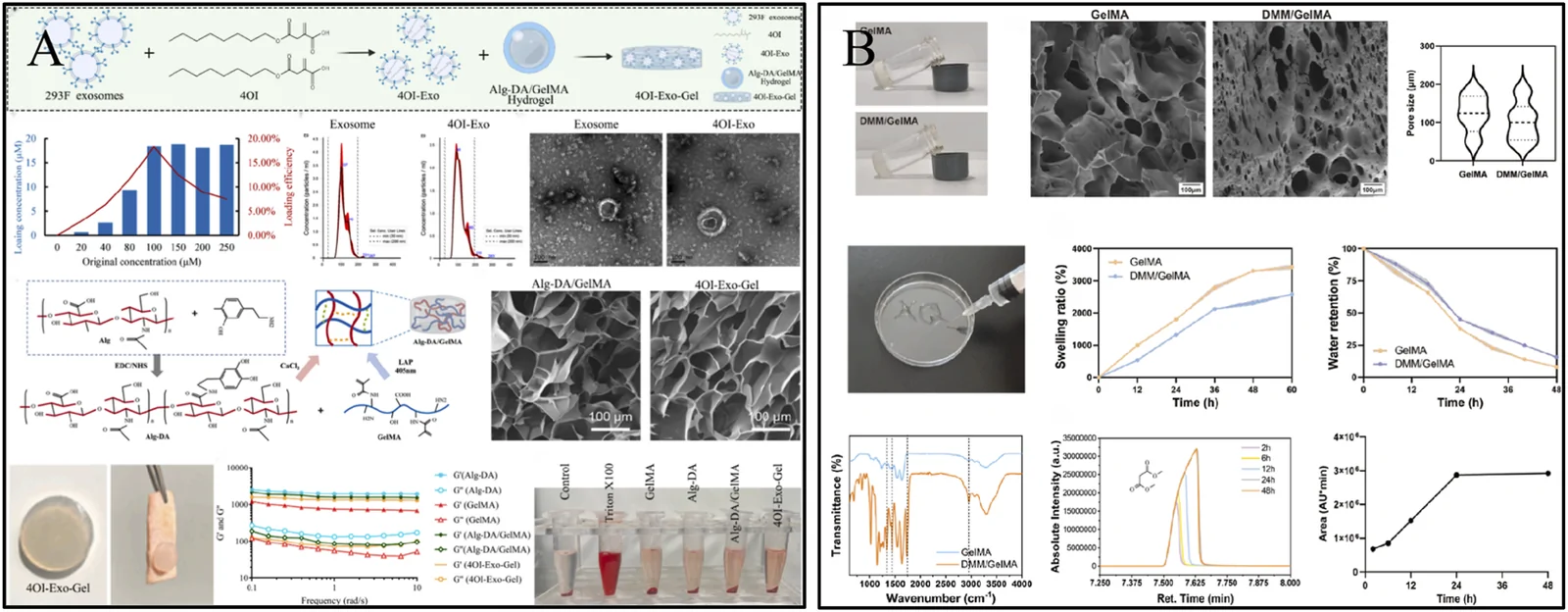
The Construction Process and Structure of Representative Hydrogels Targeting Macrophage Metabolic Dysfunction. **(A)** 4OI loaded into 293F exosomes and encapsuled into Alg-DA/GelMA hydrogels. Reproduced from Shen et al. (2024). Copyright 2024 Elsevier. **(B)** Characterization of GelMA and DMM/GelMA hydrogels. Reproduced from Shao et al. (2025). Copyright 2024 Elsevier.

To disrupt macrophages’ reliance on glycolysis, researchers have turned their attention to endogenous metabolic regulators. For example, itaconate and its derivatives, such as 4-octyl itaconate (4-OI), have been identified as core switches for macrophage metabolic reprogramming (O'Neill and Artyomov, 2019). Based on this, Shen et al. (2024) developed a stimulus-responsive hydrogel delivery system loaded with 4-OI (Figure 6A). When applied to DFU wounds, this hydrogel continuously releases 4-OI into macrophages. 4-OI can force cells to reduce glycolysis rates by inhibiting key enzymes in the glycolysis pathway, such as glyceraldehyde-3-phosphate dehydrogenase (GAPDH). Simultaneously, it strongly activates the Nrf2 antioxidant pathway, repairing impaired mitochondrial function (Tu et al., 2025). This dual intervention of “inhibiting glycolysis + protecting mitochondria” reduces pro-inflammatory metabolic features and promotes repair-associated macrophage functions. As downstream repair outcomes, this metabolic shift significantly accelerates angiogenesis and granulation tissue proliferation in diabetic mouse wounds.

Beyond inhibiting glycolysis at the cytoplasmic level, directly targeting abnormal metabolic enzymes in the mitochondrial respiratory chain represents another critical pathway for metabolic reprogramming. In the inflammatory microenvironment of DFU, succinate dehydrogenase (SDH) in macrophage mitochondria becomes hyperactive, leading to abnormal accumulation of the pro-inflammatory metabolite succinate and driving persistent reactive oxygen species (ROS) storms, thereby locking macrophages into the M1 pro-inflammatory phenotype. Dimethyl malonate (DMM), a specific competitive inhibitor of SDH, can precisely disrupt this pathological metabolic flow. However, free small-molecule drugs are easily lost in the complex wound exudate, making it difficult to maintain effective local intervention concentrations. Therefore, controlled therapeutic delivery requires not only hydrogel-mediated local retention but also rational optimization of carrier size, loading efficiency, release kinetics, and formulation parameters (Shubhra et al., 2014). To address this, Shao et al. (2025) designed and developed an injectable gelatin methacryloyl (GelMA) smart hydrogel for the localized, long-term, and sustained delivery of DMM (Figure 6B). This minimally invasive delivery system continuously supplies metabolic inhibitors to wound macrophages, blocking excessive ROS production at its source by targeting and inhibiting SDH activity on the inner mitochondrial membrane. This precise correction of the mitochondrial energy metabolism hub alleviates succinate-driven inflammatory signaling and reshapes macrophage metabolic trajectories toward a repair-supportive state, ultimately accelerating angiogenesis and epithelialization in DFU.

### 5.5 Hydrogel delivery system targeting fibroblast metabolic dysfunction

During the proliferative and remodeling phases of wound healing, fibroblasts, as the core drivers of ECM synthesis and granulation tissue growth, directly determine the efficiency of wound closure (Zhou et al., 2026). However, in diabetic patients, prolonged exposure to a metabolic disorder characterized by hyperglycemia and high oxidative stress renders fibroblasts highly susceptible to entering a state of “metabolic exhaustion” and “excessive senescence,” manifesting as loss of proliferative and migratory capacity and impaired collagen synthesis (Wu et al., 2025). To address this pathological barrier, emerging hydrogel delivery systems are achieving precise regulation of fibroblast function through strategies of “self-regulating responsiveness” and “multidimensional microenvironmental synergy”.

**FIGURE 7:**
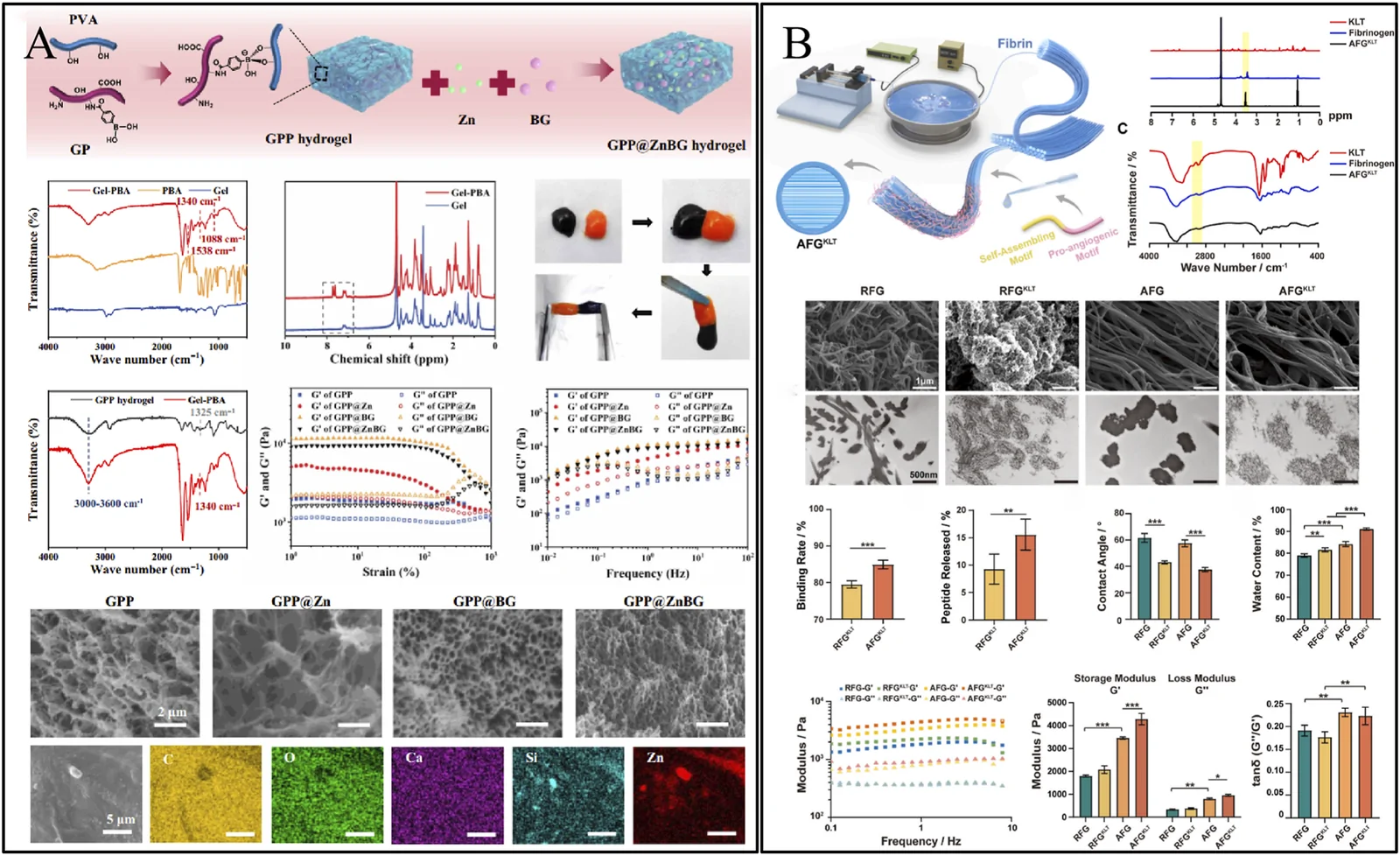
The Construction Process and Structure of Representative Hydrogels Regulating Fibroblast Dysfunction in a Metabolically Impaired DFU Microenvironment. **(A)** Preparation and characterization of GPP@ZnBG hydrogels. Reproduced from Zhao et al. (2026b). Copyright 2026 AAAS. **(B)** Fabrication and characterization of AFG^KLT^ hydrogel. Reproduced from Kim et al. (2025). Copyright 2025 KeAi.

Within the complex pathological network of DFU, the metabolic abnormalities of fibroblasts often form a vicious cycle with the wound’s hyperglycemic and high ROS environment (Zheng et al., 2026). Zhao et al. (2026b) recently developed a bioactive self-regulating hydrogel (GPP@ZnBG) capable of sensitively detecting the abnormally elevated glucose concentration and oxidative stress levels in DFU wounds (Figure 7A). Its core mechanism lies in utilizing a cascade reaction involving GOx and catalase (CAT) to consume excess wound glucose while enabling self-pH regulation and sequential release of therapeutic ions such as zinc (Zn), calcium (Ca), and silicon (Si). Single-cell RNA sequencing (scRNA-seq) results provided in-depth insights into the system’s remodeling effect on fibroblast metabolic trajectories: the released ionic flow can precisely regulate the NF-κB signaling pathway within fibroblasts, significantly reducing metabolic stress-induced inflammatory responses, and enhancing collagen deposition and granulation tissue remodeling. This closed-loop strategy, from “metabolic sensing” to “signal intervention,” successfully reactivates fibroblasts from the functional stagnation caused by metabolic impairment.

Beyond chemical signal regulation, physical structure-induced improvements in cell metabolism and behavior also demonstrate significant value. Kim et al. (2025) reported a nanofiber hydrogel with an anisotropic structure. By mimicking the oriented architecture of the natural extracellular matrix, this system provides crucial physical guidance for fibroblast migration and proliferation (Figure 7B). Addressing the cellular dysfunction in DFU wounds caused by hyperglycemia and high ROS, this hydrogel effectively alleviates the metabolic stress on fibroblasts through a “triadic synergy” involving the immune, vascular, and neural microenvironments. Compared with GPP@ZnBG, which directly responds to glucose and oxidative stress, the anisotropic nanofiber hydrogel reported by Kim et al. should be regarded as a metabolism-supportive structural strategy rather than a direct metabolic regulator. Its primary function is to mimic the oriented architecture of the extracellular matrix, guide fibroblast migration, and improve cellular behavior under hyperglycemic and oxidative stress conditions. Therefore, this system was retained in this section only as an example of how biomimetic physical cues can indirectly support fibroblast recovery in a metabolically impaired wound microenvironment.

Collectively, these examples illustrate two different levels of intervention in fibroblast dysfunction: GPP@ZnBG directly regulates the hyperglycemic and oxidative metabolic microenvironment through GOx/CAT-mediated glucose consumption and ion-mediated signaling, whereas anisotropic nanofiber hydrogels mainly provide structural support that indirectly improves fibroblast behavior under metabolic stress. This distinction avoids overstating structural guidance as direct metabolic targeting.

### 5.6 Hydrogel delivery system targeting endothelial cell metabolic dysfunction

Vascular endothelial cells (ECs) are critical components in constructing neovascular networks in wounds. In DFU, oxidative stress and metabolic disorders induced by hyperglycemic environments lead to severe endothelial dysfunction, characterized by reduced endothelial cell migration, destabilized angiogenic signaling, and abnormal cell death (Lin et al., 2025; Yang et al., 2024). To address the metabolic impairments in endothelial cells, emerging hydrogel systems offer innovative solutions for restoring wound blood supply by remodeling physical-biochemical signaling pathways and blocking pathological metabolic routes (Reid and Zhao, 2014).

**FIGURE 8:**
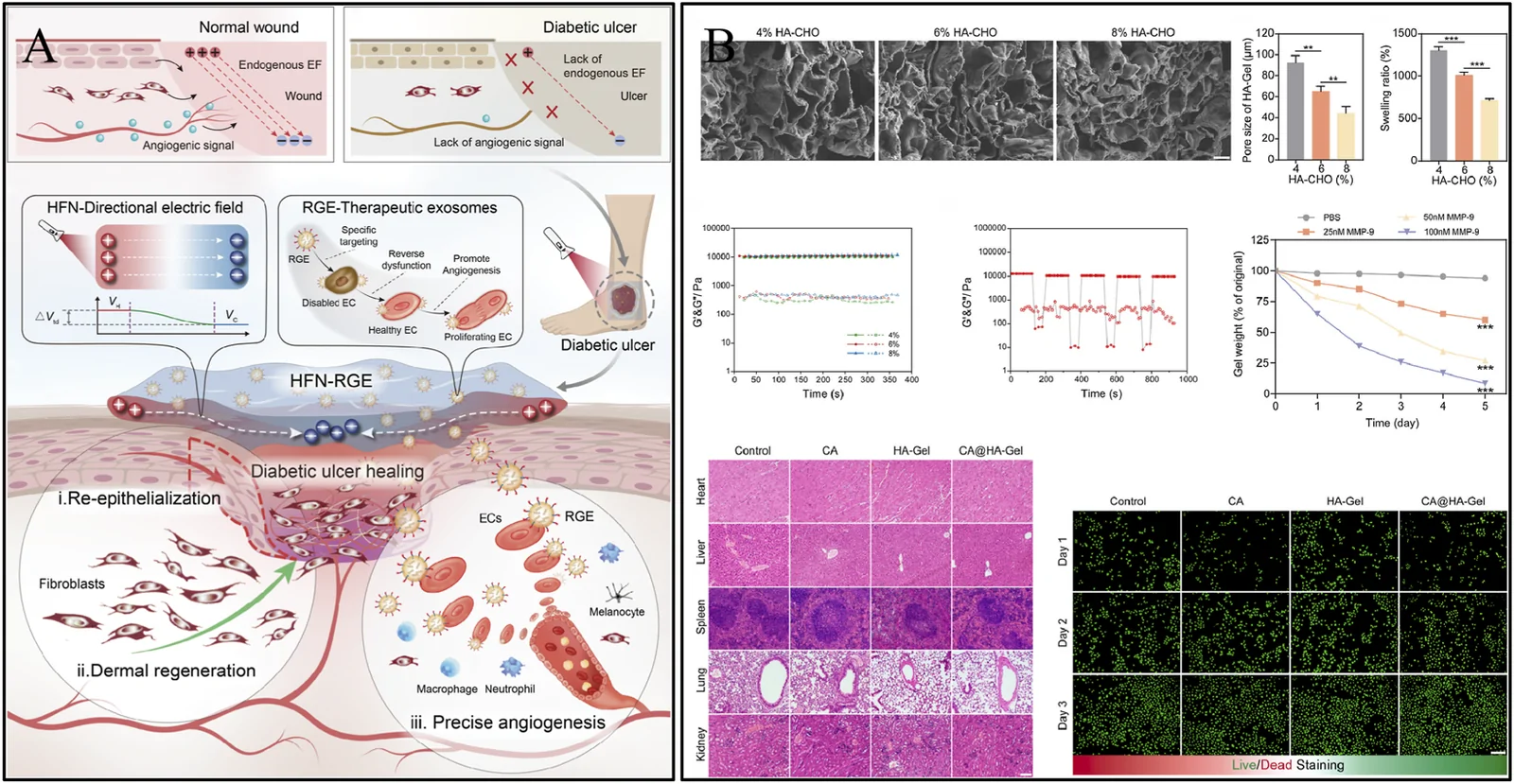
The Construction Process and Structure of Representative Hydrogels Regulating Endothelial Dysfunction and Metabolism-Associated Cell Death in DFU. **(A)** P Schematic illustration for the design and therapeutic program of the HFN-RGE. Reproduced from Tan et al. (2025). Copyright 2025 Wiley. **(B)** Characterization of HA-Gel hydroge. Reproduced from Lin et al. (2025). Copyright 2026 KeAi.

In DFU wounds, endothelial cell damage often stems from the loss of endogenous physical signals (such as electric fields) and metabolic polarization of biochemical signals. Tan et al. (2025) developed a biomimetic hydrogel with wireless thermoelectric effects (HFN-RGE), composed of biodegradable hyaluronic acid co-crosslinked with poloxamer F127 (Figure 8A). Its core innovation lies in reconstructing the missing biomimetic endogenous electric field (EF) at the wound site through a “cation trap” effect, thereby triggering directional electrotaxis in endothelial cells. Furthermore, the hydrogel incorporates targeted modified ginseng-derived exosomes (RGE), which, through the synergistic action of physical electrical stimulation and biochemical signals, improves endothelial dysfunction under hyperglycemic stress, although its primary mechanism is physical-biochemical signal reconstruction rather than direct modulation of a defined metabolic pathway, significantly enhancing their proliferation and tube-forming capabilities, thereby providing robust pro-angiogenic signals for ischemic wounds.

Beyond signal reconstruction, blocking ferroptosis—a specific cell death pathway induced by metabolic stress—is another critical approach to rescuing endothelial cell function (Jiang et al., 2024; Aijun et al., 2026). In the high oxidative stress microenvironment of DFU wounds, endothelial cells often undergo ferroptosis due to lipid peroxide accumulation, leading to complete stagnation of angiogenesis. Lin et al. (2025) designed an MMP-9-responsive smart hydrogel system that utilizes pathologically overexpressed MMP-9 in DFU wounds as a trigger switch (Figure 8B). Upon sensing MMP-9, the hydrogel undergoes responsive degradation, precisely releasing anti-ferroptosis active agents (such as specific nanozymes or drugs). Studies have shown that this system significantly downregulates the expression of ferroptosis markers by modulating the redox balance within endothelial cells, thereby protecting damaged endothelial cells at the metabolic level and ensuring the continuity and stability of the angiogenesis process. In contrast to HFN-RGE, the MMP-9-responsive anti-ferroptosis hydrogel has a clearer metabolism-associated mechanism because it targets lipid-peroxidation-driven ferroptosis, a form of metabolic cell death that directly impairs endothelial survival and angiogenesis in DFU wounds.

## 6 Summary and outlook

Metabolic dysfunction is a central mechanism connecting systemic diabetes with the local failure of DFU wound healing. Persistent hyperglycemia disrupts the TCA cycle, activates the polyol pathway, and promotes AGEs, pseudohypoxia, and oxidative stress. Lipid metabolic disorders further induce lipid peroxidation, mitochondrial injury, impaired fatty acid oxidation, and ferroptosis, whereas amino acid and protein metabolic abnormalities disturb insulin signaling, arginine utilization, collagen-precursor availability, and extracellular matrix turnover. These abnormalities converge on macrophages, fibroblasts, and endothelial cells, resulting in persistent inflammation, insufficient matrix deposition, and defective angiogenesis. Hydrogel delivery systems may interrupt this pathological network through glucose-responsive release, catalytic glucose consumption, oxygen generation, lipid-peroxide scavenging, immunometabolic regulation, and support for matrix and vascular regeneration.

Despite encouraging experimental results, most metabolism-targeted hydrogel systems remain at the preclinical stage. Current animal models do not fully reproduce the combined infection, ischemia, neuropathy, mechanical stress, and metabolic heterogeneity observed in clinical DFU. Moreover, improvements in wound closure alone are insufficient to demonstrate direct metabolic regulation. Future studies should incorporate metabolomics, metabolic-flux analysis, mitochondrial-respiration assays, and cell-specific metabolic markers. Standardized manufacturing, controllable degradation, long-term immune compatibility, enzyme and nanomaterial stability, and biosafety also require systematic evaluation. Overall, metabolism-targeted hydrogels represent a promising therapeutic direction, but their clinical translation will depend on rigorous mechanistic validation and confirmation in clinically relevant DFU models.

## References

[B1] Aijun G. Hanbin D. Ju M. Yangyang B. Meiting J. Xin C. (2026). Ferroptosis in infected diabetic wounds and targeted therapeutic strategies: a narrative review. J. Acute Dis. 15 (1), 7. 10.4103/jad.jad_147_25

[B2] Aitcheson S. M. Frentiu F. D. Hurn S. E. Edwards K. Murray R. Z. (2021). Skin wound healing: normal macrophage function and macrophage dysfunction in diabetic wounds. Molecules 26, 4917. 10.3390/molecules26164917 34443506 PMC8398285

[B3] Armstrong D. G. Boulton A. J. M. Bus S. A. (2017). Diabetic foot ulcers and their recurrence. N. Engl. J. Med. 376, 2367–2375. 10.1056/NEJMra1615439 28614678

[B4] Armstrong D. G. Tan T. W. Boulton A. J. M. Bus S. A. (2023). Diabetic foot ulcers: a review. Jama 330, 62–75. 10.1001/jama.2023.10578 37395769 PMC10723802

[B5] Arnold P. K. Finley L. W. S. (2023). Regulation and function of the Mammalian tricarboxylic acid cycle. J. Biol. Chem. 299, 102838. 10.1016/j.jbc.2022.102838 36581208 PMC9871338

[B6] Balestri F. Moschini R. Mura U. Cappiello M. Del Corso A. (2022). In search of differential inhibitors of aldose reductase. Biomolecules 12, 485. 10.3390/biom12040485 35454074 PMC9024650

[B7] Bandyk D. F. (2018). The diabetic foot: pathophysiology, evaluation, and treatment. Semin. Vasc. Surg. 31, 43–48. 10.1053/j.semvascsurg.2019.02.001 30876640

[B8] Bloomgarden Z. (2018). Diabetes and branched-chain amino acids: what is the link? J. Diabetes 10, 350–352. 10.1111/1753-0407.12645 29369529

[B9] Boniakowski A. E. Kimball A. S. Jacobs B. N. Kunkel S. L. Gallagher K. A. (2017). Macrophage-mediated inflammation in normal and diabetic wound healing. J. Immunol. 199, 17–24. 10.4049/jimmunol.1700223 28630109

[B10] Chen D. Liu C. Kang Z. (2021). Thin copper hybrid structures by spray-assisted layer by layer chemical deposition on fabric surfaces for electromagnetic interference shielding. Colloid Interface Sci. Commun. 40, 100365. 10.1016/j.colcom.2021.100365

[B11] Clayton S. M. Shafikhani S. H. Soulika A. M. (2024). Macrophage and neutrophil dysfunction in diabetic wounds. Adv. Wound Care (New Rochelle) 13, 463–484. 10.1089/wound.2023.0149 38695109 PMC11535468

[B12] De Bandt J.-P. Coumoul X. Barouki R. (2023). Branched-chain amino acids and insulin resistance, from protein supply to diet-induced obesity. Nutrients 15, 68. 10.3390/nu15010068 PMC9824001 36615726

[B13] Du L. Lin C. Hu H. Zhao Y. Liao J. Al-Smadi F. (2026). Recent advances and challenges in hydrogel-based delivery of immunomodulatory strategies for diabetic wound healing. Theranostics 16, 516–544. 10.7150/thno.117949 41328337 PMC12665143

[B14] Eid S. Sas K. M. Abcouwer S. F. Feldman E. L. Gardner T. W. Pennathur S. (2019). New insights into the mechanisms of diabetic complications: role of lipids and lipid metabolism. Diabetologia 62, 1539–1549. 10.1007/s00125-019-4959-1 31346658 PMC6679814

[B15] Felig P. Marliss E. Cahill G. F. JR (1969). Plasma amino acid levels and insulin secretion in obesity. N. Engl. J. Med. 281, 811–816. 10.1056/NEJM196910092811503 5809519

[B16] Feng H. Huang W. Zhou Q. Liu T. Li H. Yue R. (2023). Efficacy and safety of resina draconis for wound repair in the treatment of diabetic foot ulcer: a systematic review and meta-analysis of randomized controlled trials. Complement. Ther. Clin. Pract. 50, 101707. 10.1016/j.ctcp.2022.101707 36402062

[B17] Fu K. Zheng X. Chen Y. Wu L. Yang Z. Chen X. (2022). Role of matrix metalloproteinases in diabetic foot ulcers: potential therapeutic targets. Front. Pharmacol. 13, 1050630. 10.3389/fphar.2022.1050630 36339630 PMC9631429

[B18] Gaede P. Vedel P. Larsen N. Jensen G. V. Parving H. H. Pedersen O. (2003). Multifactorial intervention and cardiovascular disease in patients with type 2 diabetes. N. Engl. J. Med. 348, 383–393. 10.1056/NEJMoa021778 12556541

[B19] Gong H. Yang L. Li Y. Zhang X. Zheng C. Gan T. (2025). Metal-polyphenol nanocomposite hybrid hydrogel: a multifunctional platform for treating diabetic foot ulcers through metabolic microenvironment reprogramming. Biomaterials 322, 123414. 10.1016/j.biomaterials.2025.123414 40398214

[B20] Hehenberger K. Heilborn J. D. Brismar K. Hansson A. (1998). Inhibited proliferation of fibroblasts derived from chronic diabetic wounds and normal dermal fibroblasts treated with high glucose is associated with increased formation of l-lactate. Wound Repair Regen. 6, 135–141. 10.1046/j.1524-475x.1998.60207.x 9776856

[B21] Hellmann J. Tang Y. Spite M. (2012). Proresolving lipid mediators and diabetic wound healing. Curr. Opin. Endocrinol. Diabetes Obes. 19, 104–108. 10.1097/MED.0b013e3283514e00 22374140 PMC4038027

[B22] Hu H. Xu F. J. (2020). Rational design and latest advances of polysaccharide-based hydrogels for wound healing. Biomater. Sci. 8, 2084–2101. 10.1039/d0bm00055h 32118241

[B23] Hu X. He J. Qiao L. Wang C. Wang Y. Yu R. (2024). Multifunctional dual network hydrogel loaded with novel tea polyphenol magnesium nanoparticles accelerates wound repair of MRSA infected diabetes. Adv. Funct. Mater. 34, 2312140. 10.1002/adfm.202312140

[B24] Huang S. C. Everts B. Ivanova Y. O'Sullivan D. Nascimento M. Smith A. M. (2014). Cell-intrinsic lysosomal lipolysis is essential for alternative activation of macrophages. Nat. Immunol. 15, 846–855. 10.1038/ni.2956 25086775 PMC4139419

[B25] Huang F. Lu X. Yang Y. Yang Y. Li Y. Kuai L. (2023). Microenvironment-based diabetic foot ulcer nanomedicine. Adv. Sci. (Weinh) 10, e2203308. 10.1002/advs.202203308 36424137 PMC9839871

[B26] Islam M. R. Manir M. S. Razzak M. Mamun M. A. Mortuza M. F. Islam M. J. (2024). Silk-enriched hydrogels with ROS-Scavenging dendrimers for advanced wound care. Int. J. Biol. Macromol. 280, 135567. 10.1016/j.ijbiomac.2024.135567 39288850

[B27] Jannapureddy S. Sharma M. Yepuri G. Schmidt A. M. Ramasamy R. (2021). Aldose reductase: an emerging target for development of interventions for diabetic cardiovascular complications. Front. Endocrinol. (Lausanne) 12, 636267. 10.3389/fendo.2021.636267 33776930 PMC7992003

[B28] Jiang T. Liu S. Wu Z. Li Q. Ren S. Chen J. (2022). ADSC-exo@MMP-PEG smart hydrogel promotes diabetic wound healing by optimizing cellular functions and relieving oxidative stress. Mater Today Bio 16, 100365. 10.1016/j.mtbio.2022.100365 35967739 PMC9364034

[B29] Jiang G. Jiang T. Chen J. Yao H. Mao R. Yang X. (2023). Mitochondrial dysfunction and oxidative stress in diabetic wound. J. Biochem. Mol. Toxicol. 37, e23407. 10.1002/jbt.23407 37341017

[B30] Jiang Q. Tang L. Xiao S. (2024). Relationship between ferroptosis and healing of diabetic foot ulcer: a prospective clinical study. Front. Endocrinol. (Lausanne) 15, 1412373. 10.3389/fendo.2024.1412373 39877845 PMC11772179

[B31] Jin X. Lan W. Yong W. Yuan Z. Li X. Cui X. (2025). Application and development of stimulus-responsive hydrogel biomaterials for diabetic wound healing: a literature review. Front. Med. (Lausanne) 12, 1740559. 10.3389/fmed.2025.1740559 41585269 PMC12823876

[B32] Jude E. B. Boulton A. J. Ferguson M. W. Appleton I. (1999). The role of nitric oxide synthase isoforms and arginase in the pathogenesis of diabetic foot ulcers: possible modulatory effects by transforming growth factor beta 1. Diabetologia 42, 748–757. 10.1007/s001250051224 10382596

[B33] Kang E. Y. Chen T. H. Garg S. J. Sun C. C. Kang J. H. Wu W. C. (2019). Association of statin therapy with prevention of vision-threatening diabetic retinopathy. JAMA Ophthalmol. 137, 363–371. 10.1001/jamaophthalmol.2018.6399 30629109 PMC6459113

[B34] Kim K. Yang J. Li C. Yang C. Y. Hu P. Liu Y. (2025). Anisotropic structure of nanofiber hydrogel accelerates diabetic wound healing via triadic synergy of immune-angiogenic-neurogenic microenvironments. Bioact. Mater 47, 64–82. 10.1016/j.bioactmat.2025.01.004 39877154 PMC11772153

[B35] Kwon B. Lee H. K. Querfurth H. W. (2014). Oleate prevents palmitate-induced mitochondrial dysfunction, insulin resistance and inflammatory signaling in neuronal cells. Biochim. Biophys. Acta 1843, 1402–1413. 10.1016/j.bbamcr.2014.04.004 24732014

[B36] Li M. Hou Q. Zhong L. Zhao Y. Fu X. (2021). Macrophage related chronic inflammation in non-healing wounds. Front. Immunol. 12, 681710. 10.3389/fimmu.2021.681710 34220830 PMC8242337

[B37] Li X. Wen S. Dong M. Yuan Y. Gong M. Wang C. (2023). The metabolic characteristics of patients at the risk for diabetic foot ulcer: a comparative study of diabetic patients with and without diabetic foot. Diabetes Metab. Syndr. Obes. 16, 3197–3211. 10.2147/dmso.s430426 37867628 PMC10590077

[B38] Liechty C. Hu J. Zhang L. Liechty K. W. Xu J. (2020). Role of microRNA-21 and its underlying mechanisms in inflammatory responses in diabetic wounds. Int. J. Mol. Sci. 21, 3328. 10.3390/ijms21093328 32397166 PMC7247578

[B39] Lima T. P. L. Passos M. F. (2021). Skin wounds, the healing process, and hydrogel-based wound dressings: a short review. J. Biomater. Sci. Polym. Ed. 32, 1910–1925. 10.1080/09205063.2021.1946461 34156314

[B40] Lin C. Hu Y. Lin Z. Du L. Hu Y. Ouyang L. (2025). MMP-9 responsive hydrogel promotes diabetic wound healing by suppressing ferroptosis of endothelial cells. Bioact. Mater. 43, 240–254. 10.1016/j.bioactmat.2024.09.006 39386223 PMC11461830

[B41] Liu W. Ou-Yang W. Zhang C. Wang Q. Pan X. Huang P. (2020). Synthetic polymeric antibacterial hydrogel for methicillin-resistant staphylococcus aureus-infected wound healing: Nanoantimicrobial self-assembly, Drug- and cytokine-free strategy. ACS Nano 14, 12905–12917. 10.1021/acsnano.0c03855 32946218

[B42] Liu E. Hu X. Zhang W. Xiao W. Shen Y. Luo Y. (2024). Efficacy and safety of ultrasound-assisted wound debridement in the treatment of diabetic foot ulcers: a systematic review and meta-analysis of 11 randomized controlled trials. Front. Endocrinol. (Lausanne) 15, 1393251. 10.3389/fendo.2024.1393251 38752180 PMC11094243

[B43] Louiselle A. E. Niemiec S. M. Zgheib C. Liechty K. W. (2021). Macrophage polarization and diabetic wound healing. Transl. Res. 236, 109–116. 10.1016/j.trsl.2021.05.006 34089902

[B44] Lu L. Liao J. Xu C. Xiong Y. Zhou J. Wang G. (2025). Kinsenoside-loaded microneedle accelerates diabetic wound healing by reprogramming macrophage metabolism via inhibiting IRE1α/XBP1 signaling axis. Adv. Sci. (Weinh) 12, e2502293. 10.1002/advs.202502293 40279546 PMC12245111

[B45] Ma Z. Ding Y. Ding X. Mou H. Mo R. Tan Q. (2023a). PDK4 rescues high-glucose-induced senescent fibroblasts and promotes diabetic wound healing through enhancing glycolysis and regulating YAP and JNK pathway. Cell Death Discov. 9, 424. 10.1038/s41420-023-01725-2 38001078 PMC10674012

[B46] Ma Z. Mo R. Yang P. Ding Y. Zhang H. Dong Z. (2023b). PDK4 facilitates fibroblast functions and diabetic wound healing through regulation of HIF-1α protein stability and gene expression. Faseb J. 37, e23215. 10.1096/fj.202300874rr 37737961

[B47] Mallanagoudra P. Ss M. R. Jaiswal S. Keshava Prasanna D. Seetharaman R. Palaniappan A. (2025). Progressive hydrogel applications in diabetic foot ulcer management: phase-dependent healing strategies. Polym. (Basel) 17, 2303. 10.3390/polym17172303 PMC12431415 40942222

[B48] Mills E. L. Kelly B. Logan A. Costa A. S. H. Varma M. Bryant C. E. (2016). Succinate dehydrogenase supports metabolic repurposing of mitochondria to drive inflammatory macrophages. Cell 167, 457–470.e13. 10.1016/j.cell.2016.08.064 27667687 PMC5863951

[B49] Morze J. Wittenbecher C. Schwingshackl L. Danielewicz A. Rynkiewicz A. Hu F. B. (2022). Metabolomics and type 2 diabetes risk: an updated systematic review and meta-analysis of prospective cohort studies. Diabetes Care 45, 1013–1024. 10.2337/dc21-1705 35349649 PMC9016744

[B50] Murray P. J. Allen J. E. Biswas S. K. Fisher E. A. Gilroy D. W. Goerdt S. (2014). Macrophage activation and polarization: nomenclature and experimental guidelines. Immunity 41, 14–20. 10.1016/j.immuni.2014.06.008 25035950 PMC4123412

[B51] Nashtahosseini Z. Eslami M. Paraandavaji E. Haraj A. Dowlat B. F. Hosseinzadeh E. (2025). Cytokine signaling in diabetic neuropathy: a key player in peripheral nerve damage. Biomedicines 13, 589. 10.3390/biomedicines13030589 40149566 PMC11940495

[B52] O'Neill L. A. J. Artyomov M. N. (2019). Itaconate: the poster child of metabolic reprogramming in macrophage function. Nat. Rev. Immunol. 19, 273–281. 10.1038/s41577-019-0128-5 30705422

[B53] O'Neill L. A. Pearce E. J. (2016). Immunometabolism governs dendritic cell and macrophage function. J. Exp. Med. 213, 15–23. 10.1084/jem.20151570 26694970 PMC4710204

[B54] Okonkwo U. A. Dipietro L. A. (2017). Diabetes and wound angiogenesis. Int. J. Mol. Sci. 18, 1419. 10.3390/ijms18071419 28671607 PMC5535911

[B55] Oyane A. Araki H. Nakamura M. Shimizu Y. Shubhra Q. T. H. Ito A. (2016). Controlled superficial assembly of DNA-Amorphous calcium phosphate nanocomposite spheres for surface-mediated gene delivery. Colloids Surf. B Biointerfaces 141, 519–527. 10.1016/j.colsurfb.2016.02.010 26896659

[B56] Pan Y. Chen L. Chen Y. Thomas E. R. Zhou S. Yang Y. (2025). Mitochondrial dysfunction in diabetic ulcers: pathophysiological mechanisms and targeted therapeutic strategies. Front. Cell Dev. Biol. 13, 1625474. 10.3389/fcell.2025.1625474 40917755 PMC12408512

[B57] Pop-Busui R. Ang L. Boulton A. J. M. Feldman E. L. Marcus R. L. Mizokami-Stout K. (2022). ADA clinical compendia series. Diagnosis and Treatment of Painful Diabetic Peripheral Neuropathy. Arlington (VA): American Diabetes Association © 2022 by American Diabetes Association. All rights reserved. None of the contents may be reproduced without the written permission of the American Diabetes Association. 35544662

[B58] Potenza M. A. Addabbo F. Montagnani M. (2009). Vascular actions of insulin with implications for endothelial dysfunction. Am. J. Physiol. Endocrinol. Metab. 297, E568–E577. 10.1152/ajpendo.00297.2009 19491294

[B59] Qi M. Xie Y. Zhou J. Liu C. Ding Q. Zhang F. (2026). Glucose-responsive Cascade nanozyme for controlled ROS release and bacterial carbon metabolic reprogramming in infected diabetic wounds. J. Control Release 391, 114590. 10.1016/j.jconrel.2025.114590 41482206

[B60] Qin P. Zhou P. Huang Y. Long B. Gao R. Zhang S. (2024). Upregulation of rate-limiting enzymes in cholesterol metabolism by PKCδ mediates endothelial apoptosis in diabetic wound healing. Cell Death Discov. 10, 263. 10.1038/s41420-024-02030-2 38811564 PMC11137154

[B61] Reid B. Zhao M. (2014). The electrical response to injury: molecular mechanisms and wound healing. Adv. Wound Care (New Rochelle) 3, 184–201. 10.1089/wound.2013.0442 24761358 PMC3928722

[B62] Rohlenova K. Veys K. Miranda-Santos I. De Bock K. Carmeliet P. (2018). Endothelial cell metabolism in health and disease. Trends Cell Biol. 28, 224–236. 10.1016/j.tcb.2017.10.010 29153487

[B63] Rumora A. E. Lentz S. I. Hinder L. M. Jackson S. W. Valesano A. Levinson G. E. (2018). Dyslipidemia impairs mitochondrial trafficking and function in sensory neurons. Faseb J. 32, 195–207. 10.1096/fj.201700206R 28904018 PMC6191072

[B64] Sattaputh C. Potisat S. Jongsareejit A. Krairittichai U. Pooreesathian K. (2012). Prevalence of factors predisposing to foot complication and their relation to other risks. J. Med. Assoc. Thai 95, 1013–1020. 23061304

[B65] Shao Y. Zhou X. Zhou S. Long J. Jin L. Shi X. (2025). Injectable DMM/GelMA hydrogel for diabetic wound healing via regulating mitochondrial metabolism and macrophage repolarization. Colloids Surfaces B Biointerfaces 248, 114488. 10.1016/j.colsurfb.2024.114488 39765076

[B66] Shen D. Yu H. Wang L. Khan A. Haq F. Chen X. (2020). Recent progress in design and preparation of glucose-responsive insulin delivery systems. J. Control Release 321, 236–258. 10.1016/j.jconrel.2020.02.014 32061789

[B67] Shen K. Zheng R. Yu B. Zhang H. Wang P. Zhao P. (2024). Suppression the glucose-induced ferroptosis in endothelial cells by 4OI-loading exosomes hydrogel for the treatment of diabetic foot ulcer. Chem. Eng. J. 497, 154696. 10.1016/j.cej.2024.154696

[B68] Shubhra Q. T. Kardos A. F. Feczkó T. Mackova H. HoráK D. TóTH J. (2014). Co-encapsulation of human serum albumin and superparamagnetic iron oxide in PLGA nanoparticles: part I. Effect of process variables on the mean size. J. Microencapsul. 31, 147–155. 10.3109/02652048.2013.814729 23875616

[B69] Simonson M. Boirie Y. Guillet C. (2020). Protein, amino acids and obesity treatment. Rev. Endocr. Metab. Disord. 21, 341–353. 10.1007/s11154-020-09574-5 32827096 PMC7455583

[B70] Singh N. Armstrong D. G. Lipsky B. A. (2005). Preventing foot ulcers in patients with diabetes. Jama 293, 217–228. 10.1001/jama.293.2.217 15644549

[B71] Singh M. Kapoor A. Bhatnagar A. (2021). Physiological and pathological roles of aldose reductase. Metabolites 11, 655. 10.3390/metabo11100655 34677370 PMC8541668

[B72] Soyoye D. O. Abiodun O. O. Ikem R. T. Kolawole B. A. Akintomide A. O. (2021). Diabetes and peripheral artery disease: a review. World J. Diabetes 12, 827–838. 10.4239/wjd.v12.i6.827 34168731 PMC8192257

[B73] Steinmetz A. (2008). Lipid-lowering therapy in patients with type 2 diabetes: the case for early intervention. Diabetes Metab. Res. Rev. 24, 286–293. 10.1002/dmrr.806 18273835

[B74] Stockwell B. R. Friedmann Angeli J. P. Bayir H. Bush A. I. Conrad M. Dixon S. J. (2017). Ferroptosis: a regulated cell death nexus linking metabolism, redox biology, and disease. Cell 171, 273–285. 10.1016/j.cell.2017.09.021 28985560 PMC5685180

[B75] Sun H. Saeedi P. Karuranga S. Pinkepank M. Ogurtsova K. Duncan B. B. (2022). IDF diabetes atlas: global, regional and country-level diabetes prevalence estimates for 2021 and projections for 2045. Diabetes Res. Clin. Pract. 183, 109119. 10.1016/j.diabres.2021.109119 34879977 PMC11057359

[B76] Sun D. Chang Q. Lu F. (2024). Immunomodulation in diabetic wounds healing: the intersection of macrophage reprogramming and immunotherapeutic hydrogels. J. Tissue Eng. 15, 20417314241265202. 10.1177/20417314241265202 39071896 PMC11283672

[B77] Szondi D. C. Wong J. K. Vardy L. A. Cruickshank S. M. (2021). Arginase signalling as a key player in chronic wound pathophysiology and healing. Front. Mol. Biosci. 8, 773866. 10.3389/fmolb.2021.773866 34778380 PMC8589187

[B78] Tan M. Liu Y. Wang Y. Li Y. Wu C. Jiang Z. (2025). Wireless thermoelectric hydrogel recreates biomimetic electric field and angiogenic signal accelerating diabetic ulcer repair. Adv. Funct. Mater. 35, 2425610. 10.1002/adfm.202425610

[B79] Tu M. Zou X. Tan X. Liu Y. Ge X. Hu Y. (2025). 4-Octyl itaconate promotes diabetic wound healing by enhancing pro-resolving macrophages via the Efferocytosis-MCT1-Lactate-GPR132 pathway and macrophage-independent synergistic effects. Diabetes Metab. J. 50 (4), 707–723. 10.4093/dmj.2024.0579 41183805 PMC13366132

[B80] Volpe C. M. O. Villar-Delfino P. H. Dos Anjos P. M. F. Nogueira-Machado J. A. (2018). Cellular death, reactive oxygen species (ROS) and diabetic complications. Cell Death Dis. 9, 119. 10.1038/s41419-017-0135-z 29371661 PMC5833737

[B81] Wan R. Weissman J. P. Grundman K. Lang L. Grybowski D. J. Galiano R. D. (2021). Diabetic wound healing: the impact of diabetes on myofibroblast activity and its potential therapeutic treatments. Wound Repair Regen. 29, 573–581. 10.1111/wrr.12954 34157786

[B82] Wan R. Lin Z. Xu M. Luo W. Jia H. Hu Z. (2025). An injectable hyaluronic acid-silanol hydrogel containing arginine and puerarin for immune modulation and enhanced diabetic wound healing. Bioact. Mater 54, 850–870. 10.1016/j.bioactmat.2025.08.040 41268352 PMC12628066

[B83] Wang X. Y. Hu Y. (2025). Targeted energy metabolomics study on wound tissue of diabetic rats. Zhonghua Shao Shang Yu Chuang Mian Xiu Fu Za Zhi 41, 137–144. 10.3760/cma.j.cn501225-20241014-00385

[B84] Wang T. J. Larson M. G. Vasan R. S. Cheng S. Rhee E. P. Mccabe E. (2011). Metabolite profiles and the risk of developing diabetes. Nat. Med. 17, 448–453. 10.1038/nm.2307 21423183 PMC3126616

[B85] Wang Z. Lu J. Yuan Z. Pi W. Huang X. Lin X. (2023). Natural carrier-free binary small molecule self-assembled hydrogel synergize antibacterial effects and promote wound healing by inhibiting virulence factors and alleviating the inflammatory response. Small 19, e2205528. 10.1002/smll.202205528 36446719

[B86] Wang K. Wang Y. Shi W. Shen K. Tao K. Ling R. (2024a). Diagnosis and treatment of diabetic foot ulcer complicated with lower extremity vasculopathy: consensus recommendation from the chinese medical association (CMA), chinese medical doctor association (CMDA). Diabetes Metab. Res. Rev. 40, e3776. 10.1002/dmrr.3776 38402455

[B87] Wang Z. Zhao F. Xu C. Zhang Q. Ren H. Huang X. (2024b). Metabolic reprogramming in skin wound healing. Burns Trauma 12, tkad047. 10.1093/burnst/tkad047 38179472 PMC10762507

[B88] Wang H. Yao S. Mo Q. Chen M. He D. Yan L. (2025). L-arginine-loaded microneedle patch enhances diabetic wound healing by regulating macrophage polarisation and mitochondrial homeostasis. Regen. Biomater. 12, rbaf092. 10.1093/rb/rbaf092 41048975 PMC12493038

[B89] Wu Q. Wang L. Yu H. Wang J. Chen Z. (2011). Organization of glucose-responsive systems and their properties. Chem. Rev. 111, 7855–7875. 10.1021/cr200027j 21902252

[B90] Wu J. Yang Q. Cheng H. Zheng H. Chen M. (2025). Metabolism, senescence, and natural products: new perspectives on wound healing in diabetes. Front. Nutr. 12, 1746827. 10.3389/fnut.2025.1746827 41572966 PMC12819320

[B91] Wynn T. A. Barron L. Thompson R. W. Madala S. K. Wilson M. S. Cheever A. W. (2011). Quantitative assessment of macrophage functions in repair and fibrosis. Curr. Protoc. Immunol. 14 (Unit14.22). Chapter, 93, 14.22.1–14.22.12. 10.1002/0471142735.im1422s93 21462164 PMC3109612

[B92] Xu Z. Liu G. Liu P. Hu Y. Chen Y. Fang Y. (2022). Hyaluronic acid-based glucose-responsive antioxidant hydrogel platform for enhanced diabetic wound repair. Acta Biomater. 147, 147–157. 10.1016/j.actbio.2022.05.047 35649507

[B93] Yan Z. Li R. Gao W. Yang J. Wang X. Zhang J. (2026). pH-responsive printable conductive hydrogels loaded with ApoVs promote diabetic wound repair through oxidative stress scavenging and macrophage reprogramming. Mater Today Bio 37, 102882. 10.1016/j.mtbio.2026.102882 41716336 PMC12915275

[B94] Yang D. R. Wang M. Y. Zhang C. L. Wang Y. (2024). Endothelial dysfunction in vascular complications of diabetes: a comprehensive review of mechanisms and implications. Front. Endocrinol. (Lausanne) 15, 1359255. 10.3389/fendo.2024.1359255 38645427 PMC11026568

[B95] Ye L. Jiang Y. Zhang M. (2022). Crosstalk between glucose metabolism, lactate production and immune response modulation. Cytokine Growth Factor Rev. 68, 81–92. 10.1016/j.cytogfr.2022.11.001 36376165

[B96] Yin R. He J. Bai M. Huang C. Wang K. Zhang H. (2019). Engineering synthetic artificial pancreas using chitosan hydrogels integrated with glucose-responsive microspheres for insulin delivery. Mater Sci. Eng. C Mater Biol. Appl. 96, 374–382. 10.1016/j.msec.2018.11.032 30606545

[B97] Zakaria N. F. Hamid M. Khayat M. E. (2021). Amino acid-induced impairment of insulin signaling and involvement of G-Protein coupling receptor. Nutrients 13, 2229. 10.3390/nu13072229 34209599 PMC8308393

[B98] Zhang Y. Lazzarini P. A. Mcphail S. M. Van Netten J. J. Armstrong D. G. Pacella R. E. (2020). Global disability burdens of diabetes-related lower-extremity complications in 1990 and 2016. Diabetes Care 43, 964–974. 10.2337/dc19-1614 32139380

[B99] Zhang H. Yan Z. Zhu J. Li Z. Chen L. Zheng W. (2025). Extracellular mitochondrial-derived vesicles affect the progression of diabetic foot ulcer by regulating oxidative stress and mitochondrial dysfunction. Adv. Sci. (Weinh) 12, e2407574. 10.1002/advs.202407574 39835574 PMC11904950

[B100] Zhao J. Hu Y. Liu C. Yan C. Liu Z. Chen Z. (2026a). Tissue-conforming organoselenium hydrogel with microphase-controlled acylhydrazone crosslinking kinetics expedites diabetic wound healing by inhibiting AGE-RAGE pathways. Adv. Mater 38, e72776. 10.1002/adma.72776 41830440

[B101] Zhao L. Chen S. Chen S. Sun Y. XI L. Huang S. (2026b). Self-regulating hydrogel for diabetic wound healing: from animal models to a pilot clinical study. Sci. Adv. 12, eaed4981. 10.1126/sciadv.aed4981 41920988 PMC13041770

[B102] Zheng J. Wang S. Sun J. Lv J. (2026). Dermal fibroblast senescence: the central hub of skin aging-from intrinsic dysfunction to microenvironmental remodeling. Int. J. Mol. Sci. 27, 1653. 10.3390/ijms27041653 41751788 PMC12940434

[B103] Zhou M. Shao J. Wu C. Y. Shu L. Dong W. Liu Y. (2019). Targeting BCAA catabolism to treat obesity-associated insulin resistance. Diabetes 68, 1730–1746. 10.2337/db18-0927 31167878 PMC6702639

[B104] Zhou Q. Sun W. W. Chen J. C. Zhang H. L. Liu J. Lin Y. (2022). Phenylalanine impairs insulin signaling and inhibits glucose uptake through modification of IRβ. Nat. Commun. 13, 4291. 10.1038/s41467-022-32000-0 35879296 PMC9314339

[B105] Zhou L. Hong Y. Li X. Zhang Y. Zhang L. Peng G. (2026). Mitochondrial mGPDH modulates fibroblast function in diabetic wound healing via the SIRT1-c-Myc-TGF-β1 axis. Diabetes 75, 427–440. 10.2337/db25-0539 41401074 PMC12928741

